# Pitfalls in the Detection of Volatiles Associated with Heated Tobacco and e-Vapor Products When Using PTR-TOF-MS

**DOI:** 10.1021/jasms.4c00062

**Published:** 2024-05-23

**Authors:** Noel Bielik, Daniela Correia, Kelly Rodrigues Crespo, Catherine Goujon-Ginglinger, Maya I. Mitova

**Affiliations:** PMI R&D, Philip Morris Products S.A., Quai Jeanrenaud 5, CH-2000 Neuchâtel, Switzerland

**Keywords:** PTR-MS, time-resolved analysis, online analysis, selected reagent ion, glycerin, propylene glycol

## Abstract

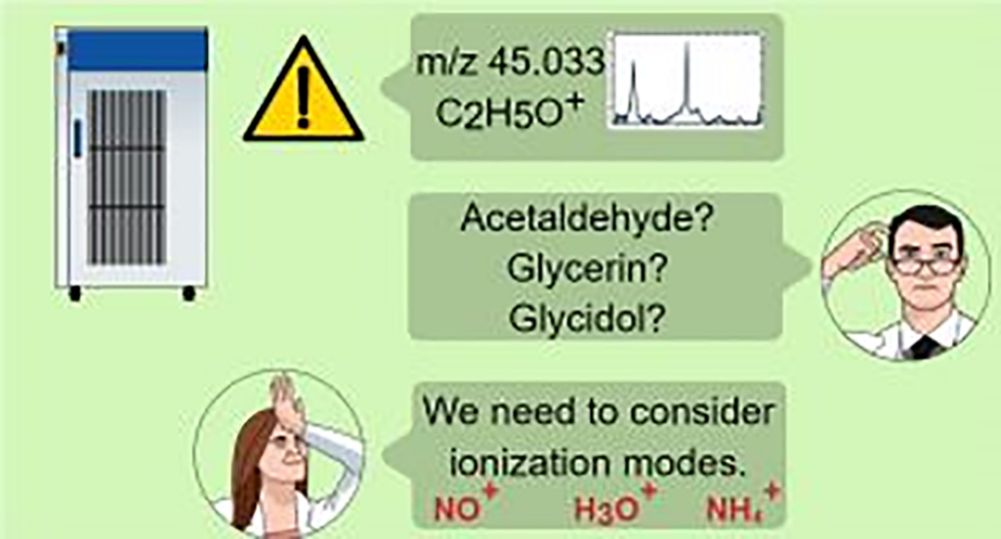

We investigated the applicability of proton transfer reaction-time-of-flight mass spectrometry (PTR-TOF-MS) for quantitative analysis of mixtures comprising glycerin, acetol, glycidol, acetaldehyde, acetone, and propylene glycol. While PTR-TOF-MS offers real-time simultaneous determination, the method selectivity is limited when analyzing compounds with identical elemental compositions or when labile compounds present in the mixture produce fragments that generate overlapping ions with other matrix components. In this study, we observed significant fragmentation of glycerin, acetol, glycidol, and propylene glycol during protonation via hydronium ions (H_3_O^+^). Nevertheless, specific ions generated by glycerin (*m*/*z* 93.055) and propylene glycol (*m*/*z* 77.060) enabled their selective detection. To thoroughly investigate the selectivity of the method, various mixtures containing both isotope-labeled and unlabeled compounds were utilized. The experimental findings demonstrated that when samples contained high levels of glycerin, it was not feasible to perform time-resolved analysis in H_3_O^+^ mode for acetaldehyde, acetol, and glycidol. To overcome the observed selectivity limitations associated with the H_3_O^+^ reagent ions, alternative ionization modes were investigated. The ammonium ion mode proved appropriate for analyzing propylene glycol (*m*/*z* 94.086) and acetone (*m*/*z* 76.076) mixtures. Concerning the nitric oxide mode, specific *m*/*z* were identified for acetaldehyde (*m*/*z* 43.018), acetone (*m*/*z* 88.039), glycidol (*m*/*z* 73.028), and propylene glycol (*m*/*z* 75.044). It was concluded that considering the presence of multiple product ions and the potential influence of other compounds, it is crucial to conduct a thorough selectivity assessment when employing PTR-TOF-MS as the sole method for analyzing compounds in complex matrices of unknown composition.

## Introduction

Direct injection mass spectrometry is a noninvasive technique facilitating the real-time analysis of many classes of compounds because of its fast response and low detection limits.^1^ Similar to other real-time measurement techniques, it has the advantage of analyzing the sample in its native state since sample preparation and storage are not required.^1,2^ Commercially available instruments based on direct injection mass spectrometry are widely used as multipurpose sensors and are applied in many fields.^1^ Among these, proton transfer reaction mass spectrometry (PTR-MS) is becoming increasingly popular because of its versatility and sensitivity in the low pptV concentration levels.^3,4^ The ionization in PTR-MS is in principal chemical ionization using proton transfer from a hydronium ion (H_3_O^+^ ion).^3^ PTR is typically coupled with time-of-flight (TOF) detectors, which enables the separation of isobaric compounds.^5^ Compared with other direct injection mass spectrometry techniques, PTR-MS has the advantage of being quantitative owing to the high degree of similarity in the ionization rate of all ionizable compounds, meaning that the instrument response is proportional to the compound quantity.^3,4^ A calibration-less quantification can be conducted by built-in algorithms using mathematical methods based on kinetic theory.^3^ This pseudoabsolute functionality improves the throughput in time-resolved measurements of gases and aerosols. The benefits and limitations of this approach are further discussed by Beauchamp et al.^6^

Use of alternative nicotine-delivery systems (e.g., heated tobacco products/heat-not-burn and e-vapor products/e-cigarettes) has increased in recent years.^7,8^ Given the relative novelty of these products, it is important to determine if they contain compounds not found in cigarette smoke. A comprehensive characterization of a heated tobacco product was performed to identify any additional constituents beyond the standard harmful and potentially harmful constituents.^9,10^ There has also been extensive investigation into certain flavors in the liquids used in e-vapor products.^11^ These studies offer the possibility of searching for potential new compounds of toxicological concern and identifying possible chemical tracers for heated tobacco and e-vapor products. In view of evaluating possible new targets, a versatile technique is needed to allow simultaneous real-time quantitative analysis of several compounds related to the use of heated tobacco and e-vapor products. Peer-reviewed evidence demonstrates that PTR-TOF-MS could be a suitable tool for such purposes.^12^

Consequently, we assessed the feasibility of real-time analysis by PTR-TOF-MS of several compounds of interest found in the aerosols of heated tobacco and e-vapor products, either in inhaled aerosol or in exhaled breath samples, focusing on the major aerosol constituents, namely, glycerin and propylene glycol, together with some of their thermal degradation products such as acetol, glycidol, and acetaldehyde and acetone, respectively. Glycerin is used in aerosol formation for heated tobacco products, while glycerin and propylene glycol typically play this role in e-vapor products. Accordingly, these compounds are major components of the aerosols of heated tobacco^13^ and e-vapor products^11^ and are present in the milligrams per item range. The presence of acetol and glycidol was reported in aerosols of heated tobacco^9^ and e-vapor products.^14^ In the aerosols of heated tobacco products, acetaldehyde and acetone were present in micrograms per item range,^15^ while their levels in aerosols of e-vapor products were typically in the nanograms per puff range with newer generations of devices with sensors preventing dry puff.^16^ Furthermore, when considering exhalation samples, it is important to remember that acetone is a major volatile organic compound in all exhaled breath samples.^17^

Glycerol and propylene glycol are considered to have low acute toxicity. The acceptable daily intake for glycerol is “not specified” due to the lack of health-related hazards,^18^ while propylene glycol has an established limit of 25 mg/kg body weight/day.^19^ Acetone, although low in acute toxicity, can nonetheless cause irritation to the throat and lungs.^20^ Limited toxicological data exist for acetol, and thus, no classification or guidance values have been set. Glycidol is considered probably carcinogenic to humans, while acetaldehyde is possibly carcinogenic.^21^ Glycerol, propylene glycol, and acetol are used as marker compounds for the aerosols of heated tobacco and e-vapor products. Acetone and acetaldehyde are listed as harmful and potentially harmful constituents in tobacco products by the U.S. Food & Drug Administration (FDA).^22^ Glycidol is included in the FDA’s list of chemicals recommended for analysis in premarket applications for e-vapor products.^23^

We investigated the selectivity of PTR-TOF-MS for time-resolved analysis of mixtures of glycerin, acetol, glycidol, acetaldehyde, acetone, and propylene glycol. Considering the elemental composition of the molecular ions and probable ion fragments, the compounds of interest were split into two groups: the first containing glycerin, acetol, glycidol, and acetaldehyde and the second containing propylene glycol and acetone. The concentration ranges for each compound were calculated based on the data for the mainstream aerosol of a popular heated tobacco product^13^ (Supplementary Table S1). Within the first group, acetol and glycidol were immediately identified as problematic for this type of analysis since they have the same elemental composition (C_3_H_6_O_2_), and hence, their protonated molecules give overlapping signals in the real-time measurement trace. It remains to be clarified if specific ions (*m*/*z*) could be identified for each compound of interest to allow time-resolved analysis of their mixtures to be conducted by PTR-TOF-MS. Hereafter, all ions are presented as theoretical exact masses for given elemental compositions. The theoretical and experimental exact masses and mass accuracies for the ions of all compounds under investigation are summarized in Supplementary Table S2.

## Methods

### Instrument Settings

The measurements were conducted on a proton transfer reaction-time-of-flight mass spectrometer (PTR-TOF-MS 6000 X2, Ionicon Analytik, Innsbruck, Austria) equipped with selective reagent ionization (SRI). Nitrogen and oxygen (synthetic air supply, N_2_ + O_2_ 20% ± 2% v/v, Carbagas, Switzerland) were mixed in the source to produce NO^+^ reagent ions. Ammonium reagent ions were generated by the reaction of synthetic air with water vapor. The sampling flow rate was 70 mL/min, and the time resolution of the PTR-TOF-MS measurements was 1000 ms. The transmission was determined on a regular basis, whenever the instrument settings were changed, or if the instrument was moved.

For the H_3_O^+^ mode, the transfer line and drift tube temperatures were maintained at 100 °C. The drift voltage was 350 V, and the drift tube pressure was 2.8 mbar. The electric field (E/N) ratio was 69 Td. The voltage across the drift tube was varied over the range from 350 to 950 V only in the experiments conducted to determine the fragmentation pattern of each compound of interest as a function of the E/N. In these cases, the values obtained for E/N ranged from 69 to 190 Td. Mass calibration in the H_3_O^+^ mode was performed before each experimental run using the following masses: *m*/*z* 21.022 (H_3_O^+^ isotope), *m*/*z* 203.943 (iodine abstraction from 1,3-diiodobenzene, [MH – I]^+^, C_6_H_5_I), and *m*/*z* 330.848 (protonated 1,3-diiodobenzene, C_6_H_5_I_2_). 1,3-Diiodobenzene was used as a built-in mass scale calibrator in PTR-TOF-MS (Permeation add-on, Ionicon Analytik, Innsbruck, Austria). During processing, these *m*/*z* values were verified, and the data was recalibrated when necessary.

In NO^+^ mode, the transfer line and drift tube temperatures were maintained at 100 °C. The drift voltage was 80 V, the drift tube pressure was 2.8 mbar, and the E/N ratio was 16 Td. Mass calibration was conducted during processing using *m*/*z* 19.018 (H_3_O^+^) and 329.840 (1,3-diiodobenzene, C_6_H_4_I_2_^+^).

In NH_4_^+^ mode, the transfer line and drift tube temperatures were maintained at 100 °C. The drift voltage was 180 V, the drift tube pressure was 2.8 mbar, and the E/N ratio was 36 Td. Mass calibration was conducted during processing using *m*/*z* 19.018 (H_3_O^+^) and 35.060 (NH_3_.NH_4_^+^).

### Chemicals

To determine the transmission of PTR-TOF-MS, a custom-made gas mix (Restek, Bellefonte, PA, USA) was used containing acetonitrile, acetone, isoprene, benzene, toluene, *o*-xylene, 1,2,4-trimethylbenzene, 1,2-dichlorobenzene, and 1,2,3-trichlorobenzene each at 1 ppm in nitrogen.

Acetaldehyde, acetol, acetone, glycidol, glycerin, and propylene glycol were purchased from Sigma-Aldrich (St. Louis, MO, USA). ^13^C_3_,D_5_-labeled glycerin, D_5_-labeled glycidol, and ^13^C_2_-labeled acetaldehyde were provided by Toronto Research Chemicals (Toronto, ON, Canada). Purified water (LC-MS Chromasolv, Thermo Fisher Scientific, Waltham, MA, USA) was used to prepare aqueous solutions of the standards.

### Standards Preparation and Measurements

The gas mix to determine the transmission was injected using a liquid calibration unit (LCU, Ionicon Analytik, Innsbruck, Austria). The sampling flow rate was adjusted to inject 5 ppb of each compound. After stabilization of the signal, the gas mix was measured for 5 min, followed by stopping the flow of the gas mix, stabilization of the signal, and then background measurement for 5 min.

For the experiments with stable isotope-labeled standards, stock solutions of individual compounds were prepared in purified water and then mixed and diluted to aqueous solutions named mixtures 1–3. Mixture 1 comprised ^13^C_3_,D_5_-labeled glycerin (89.4 μg/mL), D_5_-labeled glycidol (2.14 μg/mL), and unlabeled acetaldehyde (5.04 μg/mL). Mixture 2 contained ^13^C_3_,D_5_-labeled glycerin (93.1 μg/mL), ^13^C_2_-labeled acetaldehyde (6.64 μg/mL), and unlabeled glycidol (2.23 μg/mL). Mixture 3 was composed of ^13^C_3_,D_5_-labeled glycerin (92.5 μg/mL), D_5_-labeled glycidol (2.42 μg/mL), and unlabeled acetol (18.7 μg/mL). The LCU was used to convert mixtures 1–3 into gaseous standard mixtures. Each of these mixtures was individually injected at different dilutions to obtain a range of concentrations. The LCU temperature was 100 °C, the nitrogen flow rate was set at 1000 standard cm^3^/min, and the sample flow rate was varied for the different dilutions in the range of 0.1–10 μL/min. The flow rate of water was varied in the range of 0.1–10 μL/min depending on the dilution level, and it was adjusted to obtain a total flow rate from the two ports (sample port and water port) of 10 μL/min.

For calibration curve generation, stock solutions of individual compounds were prepared in water and then diluted: ^13^C_3_,D_5_-labeled glycerin (85.6 μg/mL), glycidol (6.74 μg/mL), D_5_-labeled glycidol (7.17 μg/mL), acetaldehyde (3.81 μg/mL), ^13^C_2_-labeled acetaldehyde (3.80 μg/mL), and acetol (15.7 μg/mL). The LCU was used for conversion of the solutions into gaseous standards. Each individual solution was injected separately at different dilutions to obtain a range of concentrations. As was the case in the previous experiment, the LCU temperature was 100 °C, the nitrogen flow rate was set at 1000 standard cm^3^/min, and the sample flow rate was varied for the different dilutions in the range of 0.1–10 μL/min. The flow rate of water was varied in the range of 0.1–10 μL/min depending on the dilution level, and it was adjusted to obtain a total flow rate from the two ports (sample port and water port) of 10 μL/min.

An LCU was used to inject all of the standards for the experiments in the different ionization modes.

Acetone and propylene glycol were introduced in gaseous form in PTR-TOF-MS using a Permeator (model V-OVG, Owlstone, Westport, CT, USA). The exit port of the Permeator was connected directly to the inlet of the PTR-TOF-MS. The permeation tube kit was purchased from Owlstone. The permeation tubes were cut to 10 cm lengths, and each was filled with 1 g of pure compound and then closed tightly. The permeation rate of each compound was determined using TD-GC-MS (thermo-desorption-gas chromatography–mass spectrometry) calibrated with authentic standards. The permeation rates were 283 ng/min at 30 °C for acetone and 130 ng/min at 100 °C for propylene glycol. The permeation tubes for the two compounds were conditioned in the oven of the Permeator (acetone at 30 °C for 2 days, propylene glycol at 100 °C for 2 days). During operation, the oven was maintained at this temperature, and the sample flow rate to the Permeator was set at 100 mL/min. To obtain the different concentration levels for the calibration curve, the flow rates were varied by changing the exhaust flow of the Permeator between 0 and 3 L/min. Each of the compounds was injected individually at different dilutions to obtain a range of different concentrations.

### FastGC-PTR-TOF-MS

The acetol, glycerin, glycidol, and propylene glycol mixture was analyzed using the fastGC add-on (version fastGC FGC230, Ionicon Analytik). The following instrumental parameters were used: NO^+^ mode, PTR drift tube: E/N 16 Td; fastGC: (a) carrier gas flow of 8.00 standard cm^3^/min and (b) temperature ramp from 50 to 180 °C in 180 s (1.38 °C/s). Then, the temperature of the GC was set to 50 °C to prepare the system for the next injection in 20 s. Nitrogen was used as a carrier and make up gas.

### Limit of Detection (LOD)

The LOD for all compounds of interest was determined based on the method proposed by Ellis and Mayhew.^3^ In case several fragments were present for a given compound, the most intense *m*/*z* was used for the calculations. The following formula was applied^3^
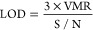
where the VMR (volume mixing ratio) and S/N (signal-to-noise) were determined for the lowest calibration standard.

## Results and Discussion

### Reactions in the Drift Tube of Glycerin, Acetol, Glycidol, and Acetaldehyde in H_3_O^+^ Mode

**Figure 1:**
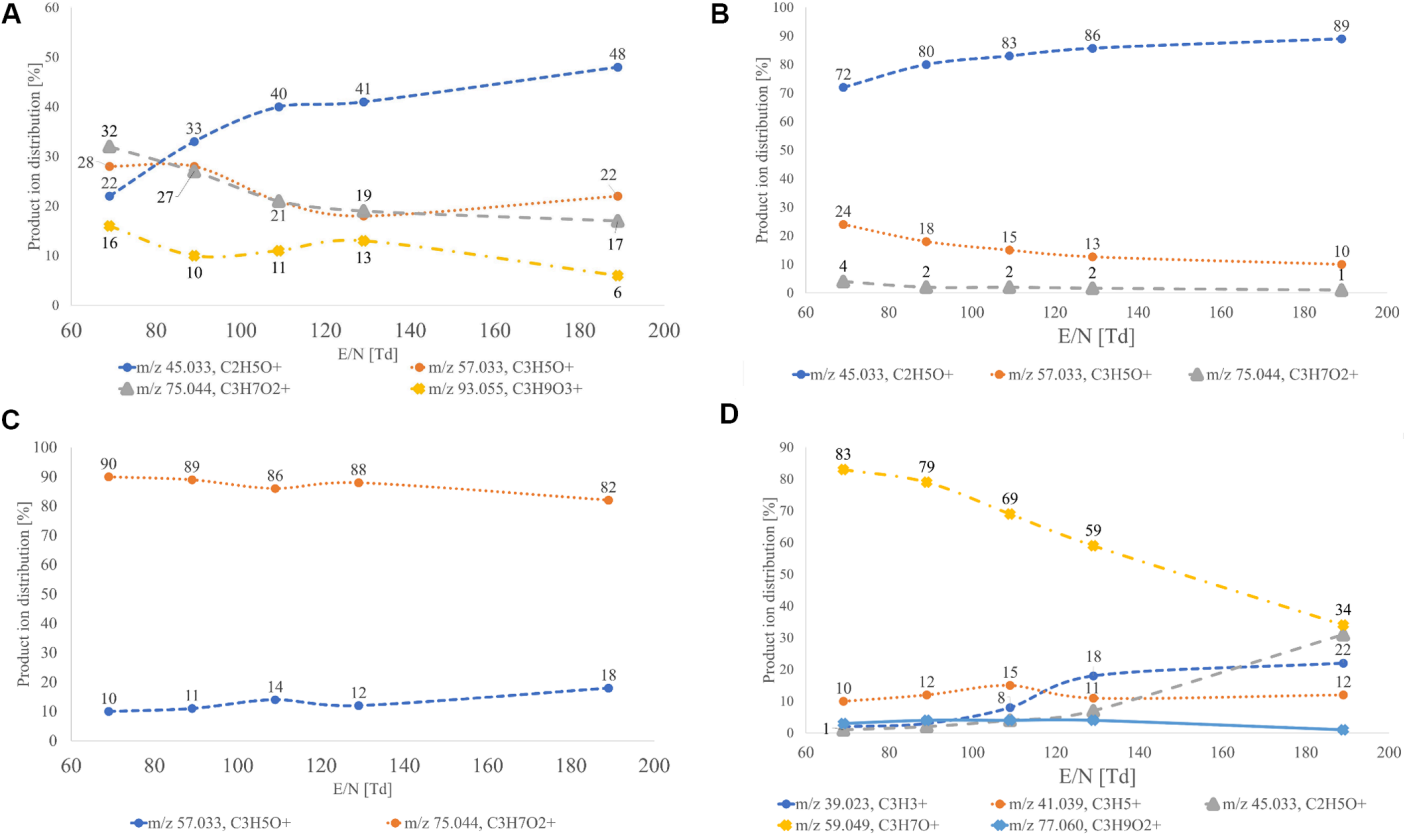
Product ion distributions (percentages) as a function of the electric field (E/N) in H_3_O^+^ mode for (A) glycerin, (B) glycidol, (C) acetol, and (D) propylene glycol.

Initially, aiming to characterize the reactions in the drift tube in the H_3_O^+^ mode of glycerin, acetol, glycidol, and acetaldehyde, the individual compounds were evaluated separately. When using H_3_O^+^ as a reagent ion, some of the most typical and dominant reaction pathways are nondissociative proton transfer yielding a protonated molecule [M + H]^+^, dissociative proton transfer leading to one or more charged fragments [F]^+^, and associative proton transfer with hydronium ion [M + H_3_O]^+^ and water clusters ([M + H·(H_2_O)_2_]^+^ and [M + H·(H_2_O)_3_]^+^).^3^ Reactions with parasitic ions (NO^+^, O_2_^+^) can also occur but to a lesser degree. However, the analysis showed no *m*/*z* coming from such reactions for any of the investigated compounds (abundances of NO^+^, O_2_^+^ relative to H_3_O^+^ < 2% in all experiments). Likewise, there were no ions for associative proton transfer with the first or second water cluster, and *m*/*z* values of only negligible intensity for associative proton transfer with hydronium ions were observed. The remaining point to clarify was whether only nondissociative proton transfer reactions were occurring in the drift tube or if undesirable dissociative proton transfer reactions were also taking place. In the latter reaction type, there is typically a decrease in analytical sensitivity due to loss of signal intensity and, possibly, a loss of selectivity resulting from overlaps with other *m*/*z*. A detailed analysis of the data yielded five important observations (Table 1, Figure 1).

- Acetaldehyde yielded a protonated molecule (*m*/*z* 45.033, C_2_H_5_O^+^),
- Glycerin, acetol, and glycidol gave protonated molecules at *m*/*z* 93.055 (C_3_H_9_O_3_^+^), *m*/*z* 75.044 (C_3_H_7_O_2_^+^), and *m*/*z* 75.044 (C_3_H_7_O_2_^+^), respectively. The protonated molecules of glycerin and glycidol were minor reaction products in the drift tube, while that of acetol was major.
- Three fragment ions of glycerin were observed in H_3_O^+^ mode (*m*/*z* 75.044, C_3_H_7_O_2_^+^, *m*/*z* 57.033, C_3_H_5_O^+^, and *m*/*z* 45.033, C_2_H_5_O^+^).
- A minor fragment ion was observed for acetol (*m*/*z* 57.033, C_3_H_5_O^+^).
- Glycidol underwent significant dissociative proton transfer reactions in H_3_O^+^ mode, leading to fragment ions at *m*/*z* 57.033 (C_3_H_5_O^+^, [M + H – H_2_O]^+^) and *m*/*z* 45.033 (C_2_H_5_O^+^, [M + H – CH_2_O]^+^).

**Table 1:** Principal Ion Species in H_3_O^+^, NO^+^, and NH_4_^+^ Modes for Acetaldehyde, Acetol, Acetone, Glycerin, Glycidol, and Propylene Glycol

compound	formula	ion formula	mechanism	*m*/*z*	% total signal
H_3_O^+^ reagent ion (H_3_O^+^ mode)^a^					
acetaldehyde	C_2_H_4_O	C_2_H_5_O^+^	[M + H] ^+^	45.033	100%
acetol	C_3_H_6_O_2_	C_3_H_7_O_2_^+^	[M + H]^+^	75.044	90%
acetol	C_3_H_6_O_2_	C_3_H_5_O^+^	[M + H – H_2_O]^+^	57.033	10%
acetone	C_3_H_6_O	C_3_H_7_O^+^	[M + H]^+^	59.049	100%
glycidol	C_3_H_6_O_2_	C_3_H_7_O_2_^+^	[M + H]^+^	75.044	4%
glycidol	C_3_H_6_O_2_	C_3_H_5_O^+^	[M + H – H_2_O]^+^	57.033	24%
glycidol	C_3_H_6_O_2_	C_2_H_5_O^+^	[M + H – CH_2_O]^+^	45.033	72%
glycerin	C_3_H_8_O_3_	C_3_H_9_O_3_^+^	[M + H]^+^	93.055	16%
glycerin	C_3_H_8_O_3_	C_3_H_7_O_2_^+^	[M + H – H_2_O]^+^	75.044	32%
glycerin	C_3_H_8_O_3_	C_3_H_5_O^+^	[M + H – 2H_2_O]^+^	57.033	28%
glycerin	C_3_H_8_O_3_	C_2_H_5_O^+^	[M + H – H_2_O–CH_2_O]^+^	45.033	22%
propylene glycol	C_3_H_8_O_2_	C_3_H_9_O_2_^+^	[M + H]^+^	77.060	3%
propylene glycol	C_3_H_8_O_2_	C_3_H_7_O^+^	[M + H – H_2_O]^+^	59.049	83%
propylene glycol	C_3_H_8_O_2_	C_2_H_5_O^+^	[M + H – CH_3_OH]^+^	45.033	1%
propylene glycol	C_3_H_8_O_2_	C_3_H_5_^+^	[M + H – 2H_2_O]^+^	41.039	10%
propylene glycol	C_3_H_8_O_2_	C_3_H_3_^+^	[M + H – 2H_2_O – 2H]^+^	39.023	2%
NO^+^ reagent ion (NO^+^ mode)					
acetaldehyde	C_2_H_4_O	C_2_H_3_O^+^	[M – H]^+^	43.018	100%
acetol	C_3_H_6_O_2_	C_3_H_6_NO_3_^+^	[M + NO]^+^	104.034	100%
acetone	C_3_H_6_O	C_3_H_6_NO_2_^+^	[M + NO]^+^	88.039	100%
glycidol	C_3_H_6_O_2_	C_3_H_6_NO_4_^+^	[M – 2H + NO + H_2_O]^+^	120.029^b^	12%
glycidol	C_3_H_6_O_2_	C_3_H_6_NO_3_^+^	[M + NO]^+^	104.034	17%
glycidol	C_3_H_6_O_2_	C_3_H_7_O_3_^+^	[M – H + H_2_O]^+^	91.039	10%
glycidol	C_3_H_6_O_2_	C_3_H_5_O_2_^+^	[M – H]^+^	73.028	62%
glycerin	C_3_H_8_O_3_	C_3_H_6_NO_4_^+^	[M – 2H + NO]^+^	120.029	47%
glycerin	C_3_H_8_O_3_	C_3_H_7_O_3_^+^	[M – H]^+^	91.039	33%
glycerin	C_3_H_8_O_3_	C_2_H_5_O_2_^+^	[M – H – CH_2_O]^+^	61.028	21%
propylene glycol	C_3_H_8_O_2_	C_3_H_6_NO_3_^+^	[M – 2H + NO]^+^	104.034	40%
propylene glycol	C_3_H_8_O_2_	C_3_H_7_O_2_^+^	[M – H]^+^	75.044	60%
NH_4_^+^ reagent ion (NH_4_^+^ mode)					
acetaldehyde	C_2_H_4_O				
acetol	C_3_H_6_O_2_	C_3_H_10_NO_2_^+^	[M + NH_4_]^+^	92.071	100%
acetone	C_3_H_6_O	C_3_H_10_NO^+^	[M + NH_4_]^+^	76.076	100%
glycidol	C_3_H_6_O_2_	C_3_H_10_NO_2_^+^	[M + NH_4_]^+^	92.071	100%
glycerin	C_3_H_8_O_3_	C_3_H_12_NO_3_^+^	[M + NH_4_]^+^	110.081	100%
propylene glycol	C_3_H_8_O_2_	C_3_H_12_NO_2_^+^	[M + NH_4_]^+^	94.086	100%

a The interpretation of the glycerin fragmentation is based on the investigation of Nimlos et al.^24^

b This ion is not a primary product. It is likely the outcome of a [MNO – 2H]^+^ ion formation, followed by subsequent association with water.

Over the range of concentrations studied, the responses of the protonated molecules and fragment ions of all compounds were linear (Supplementary Figures S1–S4). The LODs are reported in Table 2.

**Table 2:** LOD of the Compounds in H_3_O^+^, NO^+^, and NH_4_^+^ Modes

reagent ion	compound	VMR (ppbV)	S/N	LOD (ppbV)
H_3_O^+^	acetaldehyde	0.194	1.1	0.525
	acetol	0.474	12.5	0.114
	acetone	2.44	22.4	0.327
	glycerin	1.92	42.9	0.134
	glycidol	0.20	1.3	0.474
	propylene glycol	1.10	20.7	0.160
NO^+^	acetaldehyde	27.0	42.7	1.90
	acetol	6.28	19.4	0.972
	acetone	16.8	18.2	2.77
	glycerin	14.6	40.0	1.10
	glycidol	6.53	4.18	4.68
	propylene glycol	5.90	106.0	0.167
NH_4_^+^	acetone	2.16	5.7	1.14
	propylene glycol	4.19	43.6	0.288

**Figure 2:**
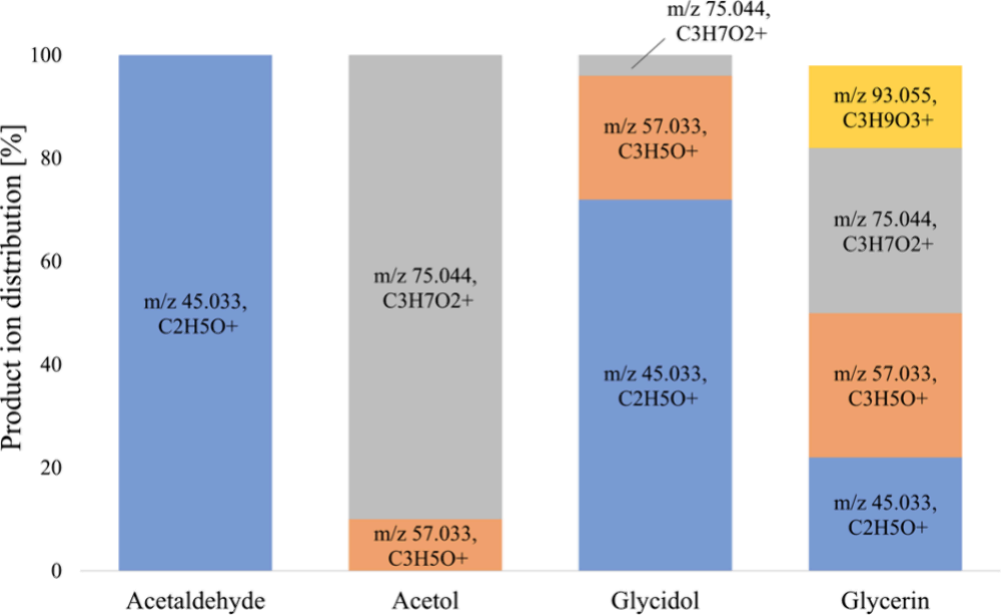
Product ion distributions in H_3_O^+^ mode at 69 Td for acetaldehyde, acetol, glycidol, and glycerin.

Thus, whenever samples containing mixtures of glycerin, acetol, glycidol, and acetaldehyde were analyzed by PTR-TOF-MS with H_3_O^+^ as the reagent ion, the following overlapping *m*/*z* values in the time-resolved trace were found (Figure 2).

- *m*/*z* 75.044 for C_3_H_7_O_2_^+^: ions for protonated acetol and glycidol and a fragment ion of glycerin [M + H – H_2_O]^+^.
- *m*/*z* 57.033 for C_3_H_5_O^+^: fragment ions of glycidol and acetol [M + H – H_2_O]^+^ and a fragment ion of glycerin [M + H – 2H_2_O]^+^.
- *m*/*z* 45.033 for C_2_H_5_O^+^: ions for protonated acetaldehyde, a fragment ion of glycidol [M + H – CH_2_O]^+^, and a fragment ion of glycerin [M + H – H_2_O – CH_2_O]^+^.

Accordingly, there was a specific ion for glycerin at *m*/*z* 93.055 (C_3_H_9_O_3_^+^), making it possible to conduct the PTR-TOF-MS analysis in H_3_O^+^ mode (Figure 2). However, it was obvious that the presence of glycerin in the sample would impact the PTR-TOF-MS analysis of the other compounds. Yet, since the dissociative proton transfer reactions of acetol and glycidol had different patterns and acetaldehyde gave a single ion, it was important to clarify if it would be possible to analyze acetol by PTR-TOF-MS in H_3_O^+^ mode using *m*/*z* 75.044, glycidol by *m*/*z* 57.033, and acetaldehyde by *m*/*z* 45.033 (Figure 2).

This investigation was carried out by applying mixtures of stable isotope-labeled compounds and unlabeled compounds. In this case, using different combinations of stable isotope-labeled compounds and unlabeled compounds in the same MS analysis made it possible to clearly distinguish the ions from different compounds within a given mixture (Supplementary Figure S5).

The accurate masses for the protonated compounds and fragments with and without a stable isotope label for each of the mixtures 1–3 used in the experiments are summarized in Table 3. The proportions of one compound versus the sums of the other compounds over the evaluated concentration range are summarized in Supplementary Tables S3–S5 for mixture 1, Supplementary Tables S6–S8 for mixture 2, and Supplementary Tables S9–S11 for mixture 3.

**Table 3:** Ions for Stable Isotope-Labeled and Unlabeled Compounds in Mixtures 1–3

ion	ion formula	.	ion formula	*m*/*z*	ion formula	*m*/*z*
mixture 1	glycerol-^13^C_3_,D_5_		glycidol-D_5_		acetaldehyde	
	^13^C_3_D_5_H_4_O_3_^+^	101.096				
ion 3	^13^C_3_D_5_H_2_O_2_^+^	83.086	C_3_D_5_H_2_O_2_^+^	80.075		
ion 2	^13^C_3_D_5_O^+^	65.075	C_3_D_5_O^+^	62.065		
ion 1	^13^C_2_D_3_H_2_O^+^	50.059	C_2_D_3_H_2_O^+^	48.052	C_2_H_5_O^+^	45.033
mixture 2	glycerol-^13^C_3_,D_5_		glycidol		acetaldehyde-^13^C_2_	
	^13^C_3_D_5_H_4_O_3_^+^	101.096				
ion 3	^13^C_3_D_5_H_2_O_2_^+^	83.086	C_3_H_7_O_2_^+^	75.044		
ion 2	^13^C_3_D_5_O^+^	65.075	C_3_H_5_O^+^	57.033		
ion 1	^13^C_2_D_3_H_2_O^+^	50.059	C_2_H_5_O^+^	45.033	^13^C_2_H_5_O^+^	47.040
mixture 3	glycerol-^13^C_3_,D_5_		glycidol-D_5_		acetol	
	^13^C_3_D_5_H_4_O_3_^+^	101.096				
ion 3	^13^C_3_D_5_H_2_O_2_^+^	83.086	C_3_D_5_H_2_O_2_^+^	80.075	C_3_H_7_O_2_^+^	75.044
ion 2	^13^C_3_D_5_O^+^	65.075	C_3_D_5_O^+^	62.065	C_3_H_5_O^+^	57.033
ion 1	^13^C_2_D_3_H_2_O^+^	50.059	C_2_D_3_H_2_O^+^	48.052		

### Influence of Glycerin, Acetol, and Glycidol on Real-Time Analysis of Acetaldehyde in H_3_O^+^ Mode

As discussed above, in PTR-TOF-MS (H_3_O^+^ reagent ion), acetaldehyde was considered for monitoring by the protonated molecule at *m*/*z* 45.033 (C_2_H_5_O^+^, hereafter ion 1). In the time-resolved trace of a given sample analyzed by PTR-TOF-MS, at this *m*/*z*, there was signal overlap for protonated acetaldehyde, the fragment [M + H – CH_2_O]^+^ of glycidol, and the fragment [M + H – H_2_O–CH_2_O]^+^ of glycerin (Figure 2). Acetol did not give any fragments at *m*/*z* 45.033. By using either mixture 1 (^13^C_3_,D_5_-labeled glycerin, D_5_-labeled glycidol, and unlabeled acetaldehyde) or mixture 2 (^13^C_3_,D_5_-labeled glycerin, ^13^C_2_-labeled acetaldehyde, and unlabeled glycidol), it was possible to evaluate the contribution of each compound to ion 1. Indeed, each of the stable isotope-labeled and unlabeled compounds generated well-resolved signals (Table 3, Supplementary Figure S5).

In mixtures with different concentrations of glycerin, glycidol, and acetaldehyde, glycerin contributed 10–99% (mixture 1, Supplementary Table S12) or 7–98% (mixture 2, Supplementary Table S13) of the signal for ion 1. In cases where the glycerin to acetaldehyde and glycidol proportion was lower than 2.5 (Supplementary Table S3) or 4.2 (Supplementary Table S6), the contribution of the glycerin fragment to ion 1 was below 50% (Supplementary Tables S12 and S13). However, if glycerin was present at a higher proportion versus acetaldehyde and glycidol, a significant portion of ion 1 was derived from the glycerin fragment (51–99% Supplementary Table S12, 51–98% Supplementary Table S13). On the other hand, at least one-half of the signal for ion 1 was attributed to acetaldehyde, where the acetaldehyde to glycerin and glycidol proportion was higher than 0.4 (Supplementary Tables S4, S7, S14, and S15). Furthermore, only at the highest investigated levels for acetaldehyde versus the lowest studied concentrations for glycerin and glycidol was the contribution of acetaldehyde to ion 1 substantial (75–92%, Supplementary Tables S14 and S15). At the concentration ranges and proportions investigated, the impact of glycidol on ion 1 was low (0.1–27%, Supplementary Tables S16 and S17), except for the highest studied levels of glycidol combined with the lowest concentrations of both acetaldehyde and glycerin, resulting in 30–52% of the signal being attributed to glycidol (Supplementary Tables S16 and S17).

### Influence of Glycerin, Acetaldehyde, and Acetol on Real-Time Analysis of Glycidol in H_3_O^+^ Mode

Three *m*/*z* values were monitored in the glycidol PTR-TOF-MS analysis in H_3_O^+^ mode: a protonated molecule at *m*/*z* 75.044, corresponding to elemental composition C_3_H_7_O_2_^+^ (hereafter Ion 3), a fragment ion resulting from neutral loss of water (*m*/*z* 57.033, C_3_H_5_O^+^, hereafter ion 2), and a fragment ion following neutral losses of CH_2_O and water (*m*/*z* 45.033, C_2_H_5_O^+^, ion 1) (Figure 1B). In the time-resolved trace of PTR-TOF-MS, there was an overlap with several other compounds at each of these *m*/*z* values (Figure 2). As previously mentioned, in the concentration range and proportions investigated for glycerin, acetaldehyde, and glycidol, the contribution of glycidol to ion 1 was low (0.152%, in most cases 0.1–15%, Supplementary Tables S16 and S17). These results clearly demonstrated that in the presence of glycerin and acetaldehyde, ion 1 was unsuitable for monitoring this compound in H_3_O^+^ mode. Initially, ion 2 was considered the best suited for the analysis of glycidol, while ion 3 was appropriate for acetol (Figure 2).

Since acetaldehyde did not generate any *m*/*z* overlapping with ion 2, it was irrelevant for this analysis. The influence of acetol on ion 2 in the presence of the other three compounds (glycerin, acetol, and glycidol) was investigated by adding nonlabeled acetol to a mixture of ^13^C_3_,D_5_-labeled glycerin and D_5_-labeled glycidol (mixture 3, Table 3). It was deduced that acetol would have a lower impact on ion 2 of glycidol, considering acetol’s very low rate of dissociative proton transfer reaction (Figure 2). In contrast, since glycerin underwent significant dissociative proton transfer (Figure 2), a strong impact of glycerin on ion 2 was assumed in samples with high glycerin levels.

When different concentrations of glycerin, acetol, and glycidol were present in the same mixture (mixture 3), glycerin impacted the intensity of ion 2 in the range of 24–99% (Supplementary Table S18), while the acetol influence was 1–73% (Supplementary Table S19). In both cases, the contribution of glycidol to ion 2 was low (0.1–24%), even at its highest investigated concentration combined with the lowest studied levels of acetol and glycerin (Supplementary Table S20). Accordingly, ion 2 was deemed inappropriate for monitoring glycidol in the studied concentration ranges of glycerin, acetol, and glycidol.

### Influence of Glycerin and Glycidol on Real-Time Analysis of Acetol in H_3_O^+^ Mode

As discussed above, acetol gave rise to two *m*/*z* in the analysis by PTR-TOF-MS with the H_3_O^+^ reagent ion: one major ion corresponding to the protonated molecule (*m*/*z* 75.044, C_3_H_7_O_2_^+^, ion 3) and a minor fragment ion resulting from neutral loss of water (*m*/*z* 57.033, C_3_H_5_O^+^, ion 2) (Figure 1C). In the time-resolved trace of PTR-TOF-MS, these two *m*/*z* of acetol overlapped with those of glycerin and glycidol (Figure 2). Given the relative ion intensities in the traces of the individual compounds, ion 2 was considered suitable for monitoring glycidol (Figure 1B) and ion 3 for monitoring acetol (Figure 1C).

The contribution of acetol to ion 3 in samples containing different levels of glycerin, acetol, and glycidol was studied in mixture 3 (nonlabeled acetol, ^13^C_3_,D_5_-labeled glycerin, and D_5_-labeled glycidol, Table 3). The results indicated that the glycerin and acetol contributions to ion 3 were in the ranges of 5–96% (Supplementary Table S21) and 4–95% (Supplementary Table S22), respectively. When the acetol to glycerin and glycidol ratio exceeded 0.7, acetol contributed to over 75% of ion 3. Moreover, the impact of glycidol on ion 3 was irrelevant (0.02–7%, Supplementary Table S23).

In the concentration range and proportions investigated, in certain conditions (acetol level equal to/higher than vs the sum of glycerin and glycidol levels), ion 3 could be used for monitoring acetol (Supplementary Table S22).

### Investigation of Alternative Reagent Ions

The outcome of the experiments described confirmed that it was not possible to perform time-resolved analysis by PTR-TOF-MS using H_3_O^+^ reagent ions for acetaldehyde, acetol, and glycidol in samples containing high levels of glycerin. Owing to the lack of selectivity and specificity for acetaldehyde, acetol, and particularly glycidol, it was not feasible from a practical perspective to conduct a time-resolved analysis of samples of unknown qualitative and quantitative composition with this approach. Therefore, alternative ionization modes of PTR-TOF-MS were investigated to identify a more selective method for time-resolved analysis of these compounds. NO^+^ and NH_4_^+^ ionization modes were explored in two separate sets of experiments. Indeed, the use of NO^+^ and NH_4_^+^ reagent ions in PTR-MS provides some selectivity for compounds with different functional groups or structural features and sometimes creates simpler spectra.^4,25,26^

Depending on the target compound, there are several dominant reaction pathways using NO^+^ as the reagent ion, specifically charge transfer [M]^+^, hydride abstraction [M – H]^+^, hydroxide abstraction [M – OH]^+^, cluster formation with NO^+^ [M + NO]^+^, and cluster formation with hydride abstraction [M + NO – 2H]^+^.^26−28^

Different ionization reactions could occur depending on compounds’ functional groups, which facilitate the analysis of compounds of identical elemental composition. For example, Koss et al.^27^ showed that using PTR-MS in NO^+^ mode permits time-resolved measurement of isomeric aldehydes and ketones in outdoor air because these carbonyls have a different ionization mechanism depending on the functional groups. Based on the compound structure, different product ions are formed in reactions with NO^+^ as the reagent ion, which ensures higher selectivity in PTR-MS analysis of gas mixtures.^25^

Regarding NH_4_^+^ ionization mode, the reaction mechanisms resemble those occurring in H_3_O^+^ mode and typically include, depending on the target compound, a nondissociative proton transfer [M + H]^+^, an associative proton transfer with ammonium ion [M + NH_4_]^+^, or an ammonium cluster [M + NH_3_ + NH_4_]^+^.^29^

Although using NH_4_^+^ as a reagent ion is not very common, it had advantages for selective time-resolved analysis in some initial experiments.^4^ Furthermore, PTR-MS in NH_4_^+^ mode was successfully applied to analyze secondary organic aerosols.^29^

The reactions in the drift tube of PTR-TOF-MS when using either NO^+^ or NH_4_^+^ reagent ions were explored in two sets of experiments for each compound. Like the experiments performed in H_3_O^+^ mode, the individual compounds (glycerin, acetol, glycidol, and acetaldehyde) were analyzed separately.

The analysis of the data in the NO^+^ mode for the compounds of interest showed, to a certain extent, improved selectivity and specificity of the method (Table 1).

- Three *m*/*z* of glycerin were observed in NO^+^ mode (*m*/*z* 120.029, C_3_H_6_NO_4_^+^, [M – 2H + NO]^+^); *m*/*z* 91.039, C_3_H_7_O_3_^+^, [M – H]^+^; *m*/*z* 61.028, C_2_H_5_O_2_^+^, [M – H – CH_2_O]^+^).
- Acetaldehyde generated a single ion (*m*/*z* 43.018, C_2_H_3_O^+^, [M – H]^+^).
- Acetol ionized via adduct formation with NO^+^ (*m*/*z* 104.034, C_3_H_6_NO_3_^+^, [M + NO]^+^).
- Glycidol yielded several reaction products at *m*/*z* 120.029 (C_3_H_6_NO_4_^+^, [M – 2H + NO + H_2_O]^+^), *m*/*z* 104.034 (C_3_H_6_NO_3_^+^, [M + NO]^+^), *m*/*z* 91.039 (C_3_H_7_O_3_^+^, [M – H + H_2_O]^+^), and *m*/*z* 73.028 (C_3_H_5_O_2_^+^, [M – H]^+^).

**Figure 3:**
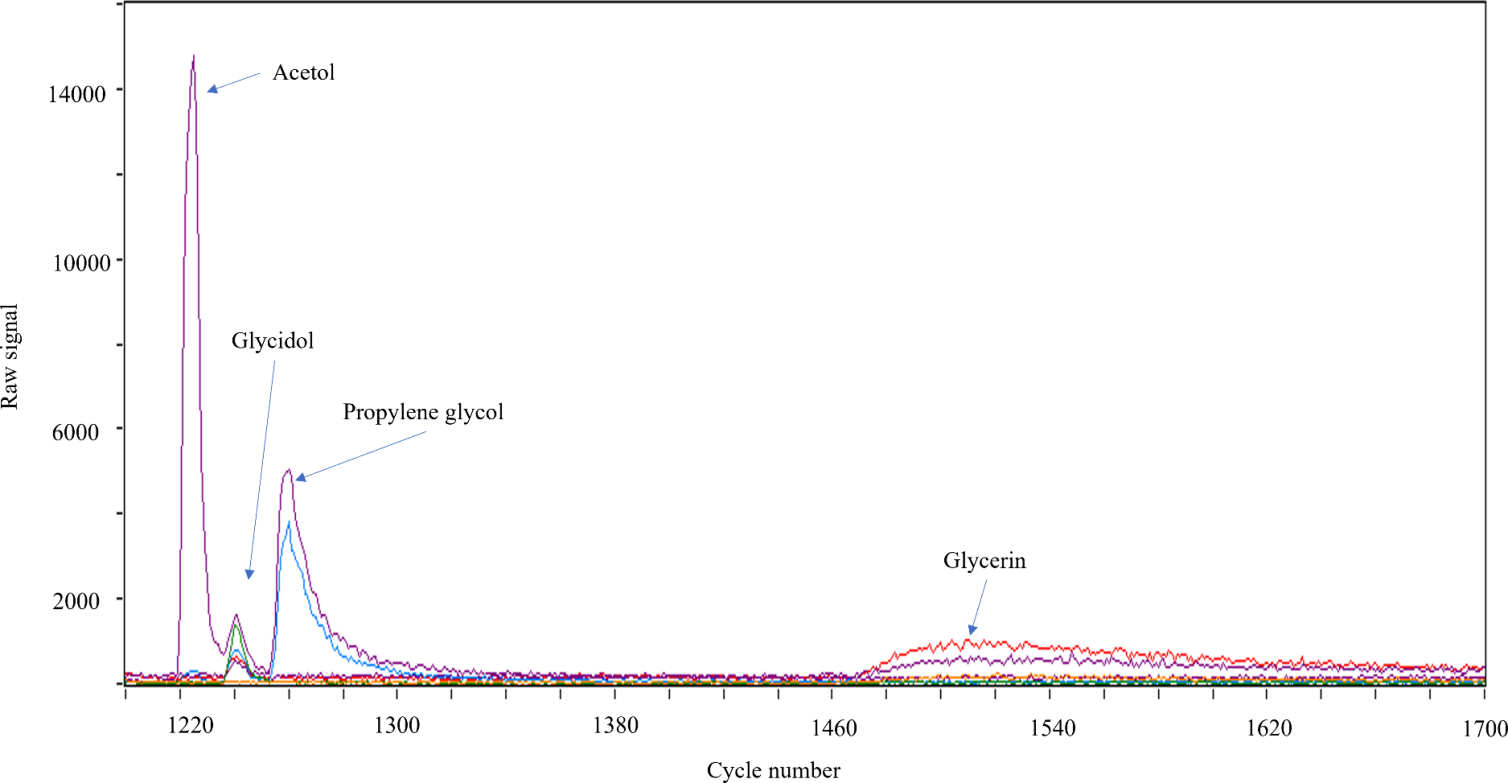
Analysis by fastGC-PTR-TOF-MS of a mixture of glycerin, glycidol, acetol, and propylene glycol in NO^+^ mode (E/N 16 Td).

Regarding the reaction products of glycidol with the NO^+^ ionic reagent, it was initially suspected that the ions at *m*/*z* 120.029 and *m*/*z* 91.039 were due to contamination of the glycidol standard. To clarify this, a mixture of glycerin and glycidol was analyzed by PTR-TOF-MS using the instrument’s fastGC add-on. Acceptable chromatographic separation of the compounds was achieved, and the product ions attributed to each of them in the initial experiment were confirmed (Figure 3).

**Figure 4:**
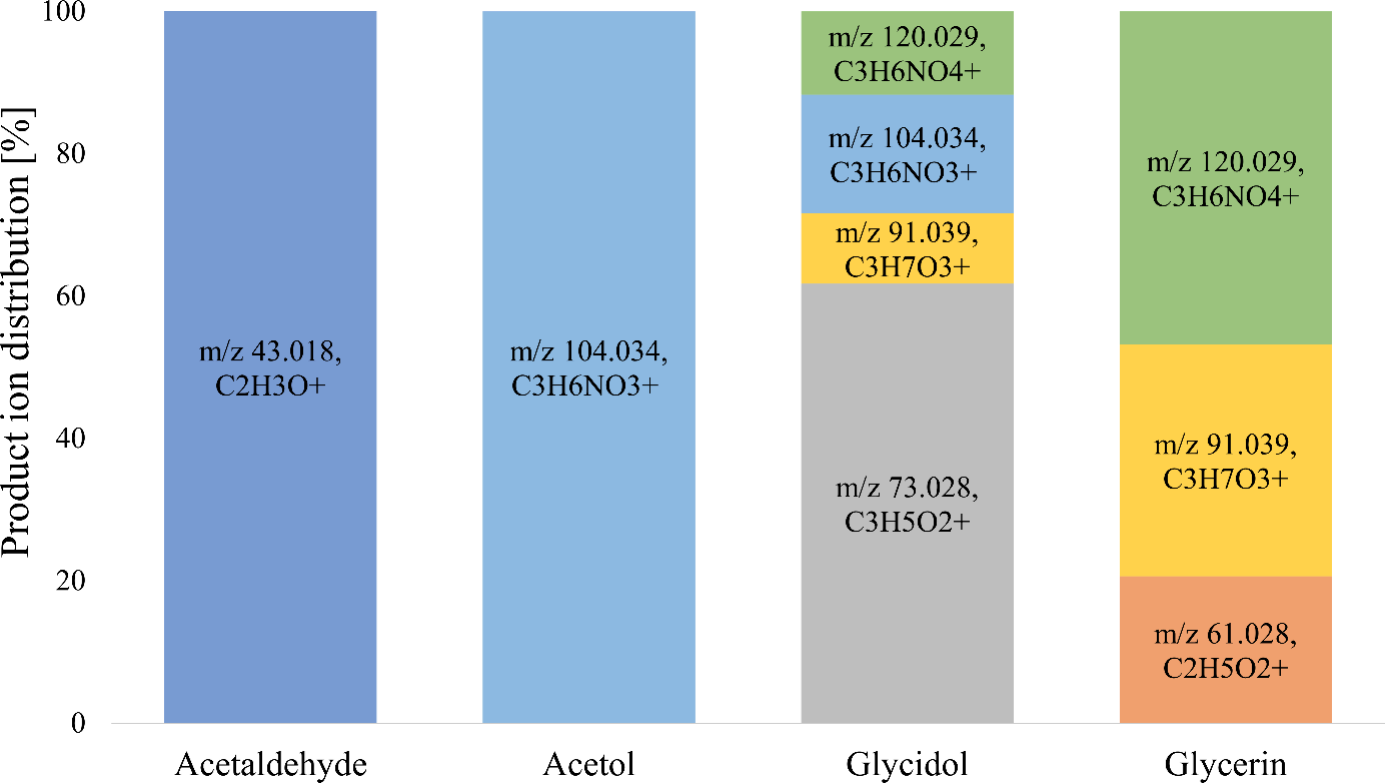
Product ion distributions in NO^+^ mode at 16 Td for acetaldehyde, acetol, glycidol, and glycerin.

The presence of several *m*/*z* for glycerin and glycidol and their overlap (*m*/*z* 120.029, C_3_H_6_NO_4_^+^, and *m*/*z* 91.040, C_3_H_7_O_3_^+^, Figure 4) indicated general issues related to selectivity in this ionization mode coupled with decreased sensitivity for both compounds. Moreover, the only ion for acetol at *m*/*z* 104.034 overlapped with the NO^+^ adduct ion of glycidol (Figure 4). These observations are of concern, especially for time-resolved analysis of complex matrices containing these compounds. Based on these findings, it would only be possible to analyze mixtures of glycerin, acetol, and glycidol in samples with known qualitative and quantitative composition. For example, in samples with glycerin and acetol as major compounds and glycidol as a minor compound, the product ions at *m*/*z* 120.029, *m*/*z* 104.034, and *m*/*z* 73.029 could provide some degree of specificity for the time-resolved analysis of glycerin, acetol, and glycidol, respectively. Concerning the application for quantitative analysis, similar to the results with the H_3_O^+^ reagent ion, the responses of the *m*/*z* in NO^+^ mode for acetaldehyde, glycerin, acetol, and glycidol were linear over the studied range of concentrations (Supplementary Figures S6–S9). The LODs are summarized in Table 2.

Furthermore, data analysis for the compounds of interest showed simpler product ion distributions in NH_4_^+^ mode compared to H_3_O^+^ mode (Table 1). Unfortunately, no ion for acetaldehyde was observed in this ionization mode. All other compounds underwent association reactions with NH_4_^+^ to generate cluster ions [M + NH_4_]^+^ at *m*/*z* 110.081 (C_3_H_12_NO_3_^+^, glycerin) and *m*/*z* 92.071 (C_3_H_10_NO_2_^+^, overlap of acetol and glycidol). These results demonstrated that the NH_4_^+^ mode was unsuitable for analyzing samples containing mixtures of acetaldehyde, acetol, glycidol, and glycerin since acetaldehyde was not ionized, while acetol and glycidol gave overlapping signals. Further experiments in NH_4_^+^ mode to evaluate linearity over the range of concentrations of interest were discontinued.

### PTR-TOF-MS Analysis of Propylene Glycol and Acetone

In view of the results discussed above on samples containing acetaldehyde, acetol, glycidol, and glycerin, it was decided to evaluate the selectivity and specificity of PTR-TOF-MS analysis for samples containing mixtures of propylene glycol and acetone directly under three different ionization modes (H_3_O^+^, NO^+^, and NH_4_^+^ reagent ions).

Three *m*/*z* were monitored in the PTR-TOF-MS analysis of propylene glycol in H_3_O^+^ mode: a minor ion for protonated molecule at *m*/*z* 77.060 (C_3_H_9_O_2_^+^), a major fragment ion resulting from neutral loss of water (*m*/*z* 59.049, C_3_H_7_O^+^), and a second minor fragment ion following neutral loss of two water molecules (*m*/*z* 41.039, C_3_H_5_^+^) (Figure 1D). At higher voltages, two other fragment ions were generated at *m*/*z* 45.033 ([M + H – CH_4_O]^+^, C_2_H_5_O^+^) and *m*/*z* 39.023 ([M + H – 2H_2_O – 2H]^+^, C_3_H_3_^+^) (Figure 1D). Similar to the behavior of glycerin in H_3_O^+^ mode, although a nondissociative proton transfer occurred, unfortunately, a significant dissociative proton transfer also took place that generated a major fragment ion. Regarding acetone, this compound underwent nondissociative proton transfer to yield an ion at *m*/*z* 59.049 (C_3_H_7_O^+^) for the protonated molecule. The signal of this ion overlapped with the fragment ion of propylene glycol. This was a similar phenomenon to that observed in experiments with the H_3_O^+^ reagent ion for glycerin, where glycerin fragment ions overlapped with the *m*/*z* of the other compounds of interest. Although this approach would be suitable for analyzing propylene glycol itself, the lack of selectivity would make it inappropriate for samples of unknown qualitative and quantitive composition. Regarding the application for quantitative analysis, the responses of the *m*/*z* in the H_3_O^+^ mode for acetone and propylene glycol were linear over the studied range of concentrations (Supplementary Figures S10 and S11). The LODs are reported in Table 2.

In NO^+^ mode, propylene glycol generated two *m*/*z* corresponding to hydride abstraction (*m*/*z* 75.044, [M – H]^+^, C_3_H_7_O_2_^+^) and formation of a cluster with NO^+^ accompanied by hydride abstraction (*m*/*z* 104.034, [M – 2H + NO]^+^, C_3_H_6_NO_3_^+^) (Table 1). The assignment of the *m*/*z* of propylene glycol was confirmed by fastGC-PTR-TOF-MS analysis (Figure 3). For acetone, this compound yielded one single ion following cluster formation with NO^+^ (*m*/*z* 88.039, C_3_H_6_NO_2_^+^, [M + NO]^+^) (Table 1). These results show that although this ionization mode would theoretically permit selective analysis by PTR-TOF-MS of mixtures of both compounds, the ion at *m*/*z* 104.034 would overlap with the adduct ions of acetol and glycidol (Table 1). Therefore, the approach was considered unsuitable for samples of unknown composition. Concerning quantitative analysis, similar to the results with the H_3_O^+^ reagent ion, the responses of *m*/*z* in the NO^+^ mode for acetone and propylene glycol were linear over the range of concentrations investigated (Supplementary Figures S12 and S13).

Analysis of the product ions of propylene glycol and acetone with the NH_4_^+^ ionic reagent showed the presence of a single *m*/*z* resulting from ionization via adduct formation with NH_4_^+^ at *m*/*z* 94.086 (propylene glycol, C_3_H_12_NO_2_^+^) and at *m*/*z* 76.076 (acetone, C_3_H_10_NO^+^) (Table 1). Thus, this ionization mode was deemed appropriate for time-resolved analysis of mixtures of propylene glycol and acetone by PTR-TOF-MS. Similar to the analysis with H_3_O^+^ and NO^+^ reagent ions, the responses of the ions in NH_4_^+^ mode for acetone and propylene glycol were linear over the range of concentrations studied (Supplementary Figures S14 and S15).

### Implication for PTR-TOF-MS Analysis

The literature contains ample evidence that a number of compounds undergo a dissociative proton transfer reaction with H_3_O^+^ ionic reagent.^3,17,30−33^ This generates identical small fragments in many cases, complicating spectra interpretation and impacting the time-resolved analysis. By using NO^+^ reagent ions, simpler spectra are obtained in some cases^26−28^ or the selectivity of the analysis is improved.^27,34^ Nevertheless, multiple product ions and/or ionic fragments could be obtained from some compounds, thereby limiting the selectivity of PTR-MS in this ionization mode.^27,34^ Thus, it is crucial to highlight that fragmentation in both H_3_O^+^ and NO^+^ modes is not only a common phenomenon but also one that is frequently observed.

Proton donation from NH_4_^+^ is less exothermic than that from H_3_O^+^, which might reduce ion fragmentation and simplify spectra.^4^ However, fewer compounds could be ionized via reaction with NH_4_^+^ reagent ions, so from a practical perspective, some authors consider the potential benefits of using alternatives to H_3_O^+^ as proton sources to be minimal.^4^ Overall, there are limitations in terms of selectivity and specificity for time-resolved studies of emissions of volatile organic compounds by PTR-MS in complex matrices. Chromatographic techniques avoid many of these limitations but have major drawbacks, such as much slower time resolution and substantial resource requirements to achieve time-resolved analysis. PTR-TOF-MS remains a powerful tool for conducting real-time quantitative analysis. The selectivity issues are less concerning for major compounds since, even if their *m*/*z* overlaps with that of another compound, a substantial portion of the ion can still be attributed to the intended compound. Nevertheless, a significant problem arises when dealing with minor compounds. In such cases, if only 10–30% of the *m*/*z* corresponds to the target compound, the analysis will likely fall within the uncertainty range of the method, rendering real-time analysis impractical. For these reasons, different research groups approach the challenges of time-resolved analysis in varied ways. For example, the time-resolved analysis of volatile organic compounds by PTR-MS is reported by presenting the results using *m*/*z* coupled to certain elemental compositions without assigning compound names.^35^ Other research groups present data by assigning typical volatile organic compounds to the measured *m*/*z*^17,36^ or by using a mixed approach with some elemental compositions combined with typical volatile organic compounds for a certain type of sample.^27,37,38^ Finally, to take advantage of the time-resolved analysis by PTR-MS and address the selectivity issue of this technique, some researchers recommend coupling this analysis at the initial stage of the study to a chromatography-based technique (e.g., GC-MS, fastGC, LC-MS).^30,32,39^ Considering the results of our study, we are fully aligned with this recommendation. Indeed, our findings demonstrate that it is necessary to couple the time-resolved analysis by PTR-MS with qualitative and quantitative evaluation of the samples by a chromatography-based technique during the initial stages. It is also crucial to carefully evaluate the most appropriate ionization mode and the behavior of the compounds in the drift tube. Applying this approach would help avoid erroneous time-resolved results. For example, if the influence of the major compound glycerin on PTR-TOF-MS (H_3_O^+^ mode) quantification of acetol, glycidol, and acetaldehyde in the aerosols of heated tobacco and e-vapor products was not considered, the quantities of all of these compounds would be overestimated. The same applies to the influence of propylene glycol on the analysis of acetone.

This has implications beyond the analyses of recreational practices such as heated tobacco and e-vapor product use. For example, glycerin and propylene glycol are used to generate artificial fog in the entertainment industry,^40^ glycerin is a widely used ingredient in toiletries,^41^ and propylene glycol is a common food additive.^19^ Accordingly, these compounds are more frequent in indoor environments and exhaled breath than could be imagined at first glance. However, it is not immediately obvious to consider possible interferences from these compounds in real-time analysis by PTR-TOF-MS (and probably other direct injection mass spectrometry techniques) when evaluating acetaldehyde as an indoor pollutant or acetaldehyde, glycidol, and acetone as exposure markers in exhaled breath. It is of interest to note that acetaldehyde and acetone were proposed as markers of disease in exhaled breath analysis.^42^ According to our results, their ions in the time-resolved trace of PTR-TOF-MS might be strongly influenced by the presence of other compounds, thus leading to erroneous results in cases where only this or other direct injection mass spectrometry techniques are used. Therefore, it is imperative to give judicious consideration to method selectivity evaluation for the specific matrix, especially in cases where complex matrices are under investigation.

## Conclusions

These experiments investigated the suitability of PTR-TOF-MS for time-resolved quantitative analysis of mixtures of glycerin, acetol, glycidol, acetaldehyde, acetone, and propylene glycol with H_3_O^+^, NH_4_^+^_,_ and NO^+^ reagent ions.

The analysis of data collected in H_3_O^+^ mode showed that there were specific *m*/*z* for glycerin and propylene glycol, but the analysis of the other compounds was impacted. Furthermore, the outcome of an experiment with stable isotope-labeled compounds showed that the analysis of acetaldehyde and acetol was feasible by PTR-TOF with H_3_O^+^ reagent ions only in samples with low concentrations of glycerin and not in samples with high levels. Moreover, the results demonstrated that H_3_O^+^ mode was not suitable at all for the analysis of glycidol at the concentration ranges investigated.

Although selective analysis by PTR-TOF-MS in NH_4_^+^ mode was possible for propylene glycol and acetone mixtures, this was not the case for acetaldehyde, acetol, and glycidol. Concerning ionization via the NO^+^ reagent ion, the compounds of interest showed simpler product ion distributions compared to the H_3_O^+^ ionic reagent, which improved selectivity. However, it should be noted that although specific *m*/*z* were identified for acetaldehyde, acetone, glycerin, glycidol, and propylene glycol, this was not the case for acetol. Furthermore, glycerin and glycidol generated multiple *m*/*z*, indicating selectivity issues in complex matrices and decreased sensitivity in cases where both compounds were present at low levels.

Overall, these experiments demonstrated that time-resolved analysis of these compounds by PTR-TOF-MS was to a certain extent possible when using combinations of different ionization modes. Concerning heated tobacco and e-vapor products, time-resolved analysis of the major compounds glycerin and propylene glycol was feasible, while that of the minor compound glycidol appeared to be impacted in all tested ionization modes. Overall, the analysis of mixtures of acetaldehyde, acetol, glycidol, glycerin, propylene glycol, and acetone in complex matrices appeared to be ambiguous.

We have not investigated interferences from other matrix components of aerosols of heated tobacco and e-vapor products. Such interferences with the time-resolved analysis of the compounds investigated in this study are possible. This is a limitation of the present study and merits future investigation.

Regarding quantitative analysis by PTR-TOF-MS, the responses of the ions of the different compounds were linear over the studied concentration ranges in all cases. These results show the potential of PTR-TOF-MS for quantitative time-resolved analysis for major compounds in complex matrices. It is recommended to couple PTR-TOF-MS with a chromatography-based analysis in the initial development stages to avoid erroneous results due to the presence of isomers or isomeric fragments.

## Supporting Information Available

Table S1, Concentration ranges for glycerin, acetol, glycidol, and acetaldehyde and propylene glycol and acetone for a popular heated tobacco product.; Table S2, summary of theoretical and experimental exact masses and mass accuracies for ions of compounds under investigation; Tables S3−S11, ratio between concentration levels of the compounds under investigation; Tables S12−S17, influence of ion 1 on the analysis of compounds under investigation; Tables S18−S20, influence of ion 2 on the analysis of compounds under investigation; Table S21-S23, influence of ion 3 on the analysis of compounds under investigation; Figures S1−S4 and S6−S15, calibration plots of compounds under investigation under different ionization modes; Figure S5, mass spectrum of ion 1 in mixture 1 (PDF)
