# Supplementary material for: Pitfalls in the Detection of Volatiles Associated with Heated Tobacco and e-Vapor Products When Using PTR-TOF-MS
Source: J Am Soc Mass Spectrom. 2024 May 23;35(6):1261–71. doi: 10.1021/jasms.4c00062 (PMC11157645; doi:10.1021/jasms.4c00062)
Supplement: Supplementary file 1 — js4c00062_si_001.pdf [file js4c00062_si_001.pdf]

Supplementary Information

for

Pitfalls in the detection of volatiles associated with heated tobacco and e-vapor products when using PTR-TOF-MS

Noel Bielik, Daniela Correia, Kelly Rodrigues Crespo, Catherine Goujon-Ginglinger, and Maya I. Mitova*

PMI R&D, Philip Morris Products S.A., Quai Jeanrenaud 5, CH-2000 Neuchâtel, Switzerland

*Corresponding author: Maya I. Mitova

tel: 0041-58-2422352; email: maya.mitova@pmi.com

ORCID: 0000-0001-8626-4362

Table S1. Concentration ranges for glycerin, acetol, glycidol and acetaldehyde, and propylene glycol and acetone for a popular heated tobacco product.......3
Table S2. Summary of theoretical and experimental exact masses, and mass accuracies for ions of acetaldehyde, acetaldehyde-$^{13}C_2$, acetol, acetone, glycerin, glycerin-$^{13}C_3$,D_5, glycidol, glycidol-D_5, and propylene glycol with H_3O^+, NO^+, and NH_4^+ reagent ions.......4
Table S3. Ratio between concentration of glycerin to sum of concentrations of acetaldehyde and glycidol for Mixture 1.......6
Table S4. Ratio between concentration of acetaldehyde to sum of concentrations of glycerin and glycidol for Mixture 1.......6
Table S5. Ratio between concentration of glycidol to sum of concentrations of glycerin and acetaldehyde for Mixture 1.......7
Table S6. Ratio between concentration of glycerin to sum of concentrations of acetaldehyde and glycidol for Mixture 2.......7
Table S7. Ratio between concentration of acetaldehyde to sum of concentrations of glycerin and glycidol for Mixture 2.......8
Table S8. Ratio between concentration of glycidol to sum of concentrations of glycerin and acetaldehyde for Mixture 2.......8

Table S9. Ratio between concentration of glycerin to sum of concentrations of acetol and glycidol for Mixture 3. 9
Table S10. Ratio between concentration of acetol to sum of concentrations of glycerin and glycidol for Mixture 3. 9
Table S11. Ratio between concentration of glycidol to sum of concentrations of glycerin and acetol for Mixture 3. 10
Table S12. Influence on Ion 1. Percent glycerin fragment $[^{13}C_2D_3H_2O]^+$ vs. sum of glycerin fragment $[^{13}C_2D_3H_2O]^+$, acetaldehyde $[C_2H_5O]^+$, and glycidol fragment $[C_2D_3H_2O]^+$ in Mixture 1. 10
Table S13. Influence on Ion 1. Percent glycerin fragment $[^{13}C_2D_3H_2O]^+$ vs. sum of glycerin fragment $[^{13}C_2D_3H_2O]^+$, acetaldehyde $[^{13}C_2H_5O]^+$, and glycidol fragment $[C_2H_5O]^+$ in Mixture 2. 11
Table S14. Influence on Ion 1. Percent acetaldehyde $[C_2H_5O]^+$ vs. sum of glycerin fragment $[^{13}C_2D_3H_2O]^+$, acetaldehyde $[C_2H_5O]^+$, and glycidol fragment $[C_2D_3H_2O]^+$ in Mixture 1. 11
Table S15. Influence on Ion 1. Percent acetaldehyde $[^{13}C_2H_5O]^+$ vs. sum of glycerin fragment $[^{13}C_2D_3H_2O]^+$, acetaldehyde $[^{13}C_2H_5O]^+$, and glycidol fragment $[C_2H_5O]^+$ in Mixture 2. 12
Table S16. Influence on Ion 1. Percent glycidol fragment $[C_2D_3H_2O]^+$ vs. sum of glycerin fragment $[^{13}C_2D_3H_2O]^+$, acetaldehyde $[C_2H_5O]^+$, and glycidol fragment $[C_2D_3H_2O]^+$ in Mixture 1. 12
Table S17. Influence on Ion 1. Percent glycidol fragment $[C_2H_5O]^+$ vs. sum of glycerin fragment $[^{13}C_2D_3H_2O]^+$, acetaldehyde $[^{13}C_2H_5O]^+$, and glycidol fragment $[C_2H_5O]^+$ in Mixture 2. 13
Table S18. Influence on Ion 2. Percent glycerin fragment $[^{13}C_3D_5O]^+$ vs. sum of glycerin fragment $[^{13}C_3D_5O]^+$, acetol fragment $[C_3H_5O]^+$, and glycidol fragment $[C_3D_5O]^+$ in Mixture 3. 13
Table S19. Influence on Ion 2. Percent acetol fragment $[C_3H_5O]^+$ vs. sum of glycerin fragment $[^{13}C_3D_5O]^+$, acetol fragment $[C_3H_5O]^+$, and glycidol fragment $[C_3D_5O]^+$ in Mixture 3. 14
Table S20. Influence on Ion 2. Percent glycidol fragment $[C_3D_5O]^+$ vs. sum of glycerin fragment $[^{13}C_3D_5O]^+$, acetol fragment $[C_3H_5O]^+$, and gycidol fragment $[C_3D_5O]^+$ in Mixture 3. 14
Table S21. Influence on Ion 3. Percent glycerin fragment $[^{13}C_3D_5H_2O_2]^+$ vs. sum of glycerin fragment $[^{13}C_3D_5H_2O_2]^+$, acetol $[C_3H_7O_2]^+$, and glycidol $[C_3D_5H_2O_2]^+$ in Mixture 3. 15
Table S22. Influence on Ion 3. Percent acetol $[C_3H_7O_2]^+$ vs. sum of glycerin fragment $[^{13}C_3D_5H_2O_2]^+$, acetol $[C_3H_7O_2]^+$, and glycidol $[C_3D_5H_2O_2]^+$ in Mixture 3. 15
Table S23. Influence on Ion 3. Percent glycidol $[C_3D_5H_2O_2]^+$ vs. sum of glycerin fragment $[^{13}C_3D_5H_2O_2]^+$, acetol $[C_3H_7O_2]^+$, and glycidol $[C_3D_5H_2O_2]^+$ in Mixture 3. 16

Figure S1. Calibration plot of response (cps) versus concentration (ppbV) for acetaldehyde in the H_3O^+ mode (E/N 69 Td). NB: a second injection was performed mid-range for quality control purposes to verify the consistency of response. 17
Figure S2. Calibration plot of response (cps) versus concentration (ppbV) for glycerin in H_3O^+ mode (E/N 69 Td). NB: a second injection was performed mid-range for quality control purposes to verify the consistency of response. 17
Figure S3. Calibration plot of response (cps) versus concentration (ppbV) for acetol in H_3O^+ mode (E/N 69 Td). NB: a second injection was performed mid-range for quality control purposes to verify the consistency of response. 18
Figure S4. Calibration plot of response (cps) versus concentration (ppbV) for glycidol in H_3O^+ mode (E/N 69 Td). *NB: a second injection was performed mid-range for quality control purposes to verify the consistency of response.* 18
Figure S5. Mass spectrum of Ion 1 in Mixture 1: unlabeled acetaldehyde ($C_2H_5O^+$) and stable isotope-labeled fragments of glycerin ($^{13}C_2D_3H_2O^+$) and glycidol ($C_2D_3H_2O^+$). 19

Figure S6. Calibration plot of response (cps) versus concentration (ppbV) for acetaldehyde in NO^+ mode (E/N 16 Td). NB: a second injection was performed mid-range for quality control purposes to verify the consistency of response. 19
Figure S7. Calibration plot of response (cps) versus concentration (ppbV) for glycerin in NO^+ mode (E/N 16 Td). NB: a second injection was performed mid-range for quality control purposes to verify the consistency of response. 20
Figure S8. Calibration plot of response (cps) versus concentration (ppbV) for acetol in NO^+ mode (E/N 16 Td). NB: a second injection was performed mid-range for quality control purposes to verify the consistency of response. 20
Figure S9. Calibration plot of response (cps) versus concentration (ppbV) for glycidol in NO^+ mode (E/N 16 Td). NB: a second injection was performed mid-range for quality control purposes to verify the consistency of response. 21
Figure S10. Calibration plot of response (cps) versus concentration (ppbV) for acetone in H_3O^+ mode (E/N 69 Td). NB: a second injection was performed mid-range for quality control purposes to verify the consistency of response. 21
Figure S11. Calibration plot of response (cps) versus concentration (ppbV) for propylene glycol in H_3O^+ mode (E/N 69 Td). NB: a second injection was performed mid-range for quality control purposes to verify the consistency of response. 22
Figure S12. Calibration plot of response (cps) versus concentration (ppbV) for acetone in NO^+ mode (E/N 16 Td). NB: a second injection was performed mid-range for quality control purposes to verify the consistency of response. 22
Figure S13. Calibration plot of response (cps) versus concentration (ppbV) for propylene glycol in NO^+ (E/N 16 Td). NB: a second injection was performed mid-range for quality control purposes to verify the consistency of response. 23
Figure S14. Calibration plot of response (cps) versus concentration (ppbV) for acetone in NH_4^+ mode (E/N 36 Td). NB: a second injection was performed mid-range for quality control purposes to verify the consistency of response. 23
Figure S15. Calibration plot of response (cps) versus concentration (ppbV) for propylene glycol in NH_4^+ mode (E/N 36 Td). NB: a second injection was performed mid-range for quality control purposes to verify the consistency of response. 24

Table S1. Concentration ranges for glycerin, acetol, glycidol and acetaldehyde, and propylene glycol and acetone for a popular heated tobacco product.

Compound[1]	µg/stick[2]	µg/puff[2]	Tidal breath[3] [µg /L]	Inhaled[4] [ppbV]	Exhaled[4] [ppbV]	Exhaled[4] [ppbV]
					Retention	
					50%	90%
Acetaldehyde	219	18.3	36.5	20259	10130	2026
Acetol	1134	94.5	189	62410	31205	6241
Glycerol	4630	385	771	204878	102439	20488
Glycidol	1.31	0.11	0.22	72.0	36.0	7.20
Acetone	40.7	3.39	6.78	2856	1428	286
Propylene glycol	643	53.6	107	34444	17222	3444

[1]Data for a model heated tobacco product. [2]The term “stick” is used in the context of heated tobacco products. It refers to the consumable part of such products. The aerosol resulting from consumption of one stick typically corresponds to 12 puffs accumulated. The term “puff” refers to a certain volume of mainstream aerosol collected under standardized conditions (55 mL volume, 3 sec puff duration). [3]Volume of 0.5 L. [4]Inhaled: estimated

concentrations of inhaled aerosol of one puff of mainstream aerosol of a model product. Exhaled: estimated concentrations of exhaled gas at either 50% or 90% retention in the respiratory tract.

Table S2. Summary of theoretical and experimental exact masses, and mass accuracies for ions of acetaldehyde, acetaldehyde-$^{13}C_2$, acetol, acetone, glycerin, glycerin-$^{13}C_3$,D_5, glycidol, glycidol-D_5, and propylene glycol with H_3O^+, NO^+, and NH_4^+ reagent ions.

Reagent ion	Compound	Ion formula	Mechanism	Theoretical mass	Experimental mass	Mass accuracy [ppm]
H_3O^+	Acetaldehyde	$C_2H_5O^+$	$[M+H]^+$	45.033	45.033	0.0
	Acetaldehyde-$^{13}C_2$	$^{13}C_2H_5O^+$	$[^{13}C_2\text{-}M+H]^+$	47.040	47.039	-21.3
	Acetol	$C_3H_7O_2^+$	$[M+H]^+$	75.044	75.045	13.3
	Acetol	$C_3H_5O^+$	$[M+H\text{-}H_2O]^+$	57.033	57.032	-17.5
	Acetone	$C_3H_7O^+$	$[M+H]^+$	59.049	59.050	16.9
	Glycerin	$C_3H_9O_3^+$	$[M+H]^+$	93.055	93.053	-21.5
	Glycerin	$C_3H_7O_2^+$	$[M+H\text{-}H_2O]^+$	75.044	75.043	-13.3
	Glycerin	$C_3H_5O^+$	$[M+H\text{-}2.H_2O]^+$	57.033	57.033	0.0
	Glycerin	$C_2H_5O^+$	$[M+H\text{-}H_2O\text{-}CH_2O]^+$	45.033	45.033	0.0
	Glycerin-$^{13}C_3$,D_5	$^{13}C_3D_5H_4O_3^+$	$[^{13}C_3\text{-}M+H]^+$	101.096	101.098	19.8
	Glycerin-$^{13}C_3$,D_5	$^{13}C_3D_5H_2O_2^+$	$[^{13}C_3\text{-}M+H\text{-}H_2O]^+$	83.086	83.087	12.0
	Glycerin-$^{13}C_3$,D_5	$^{13}C_3D_5O^+$	$[^{13}C_3\text{-}M+H\text{-}2.H_2O]^+$	65.075	65.075	0.0
	Glycerin-$^{13}C_3$,D_5	$^{13}C_2D_3H_2O^+$	$[^{13}C_3\text{-}M+H\text{-}H_2O\text{-}^{13}CD_2O]^+$	50.059	50.059	0.0
	Glycidol	$C_3H_7O_2^+$	$[M+H]^+$	75.044	75.042	-26.7
	Glycidol	$C_3H_5O^+$	$[M+H\text{-}H_2O]^+$	57.033	57.032	-17.5
	Glycidol	$C_2H_5O^+$	$[M+H\text{-}CH_2O]^+$	45.033	45.033	0.0
	Glycidol-D_5	$C_3D_5H_2O_2^+$	$[M+H]^+$	80.075	80.074	-12.5
	Glycidol-D_5	$C_3D_5O^+$	$[M+H\text{-}H_2O]^+$	62.065	62.063	-32.2
	Glycidol-D_5	$C_2D_3H_2O^+$	$[M+H\text{-}CD_2O]^+$	48.052	48.052	0.0
	Propylene glycol	$C_3H_9O_2^+$	$[M+H]^+$	77.060	77.059	-13.0
	Propylene glycol	$C_3H_7O^+$	$[M+H\text{-}H_2O]^+$	59.049	59.049	0.0
	Propylene glycol	$C_2H_5O^+$	$[M+H\text{-}CH_3OH]^+$	45.033	45.034	11.1
	Propylene glycol	$C_3H_5^+$	$[M+H\text{-}2.H_2O]^+$	41.039	41.039	7.3
	Propylene glycol	$C_3H_3^+$	$[M+H\text{-}2.H_2O\text{-}2H]^+$	39.023	39.023	-4.9
NO^+	Acetaldehyde	$C_2H_3O^+$	$[M\text{-}H]^+$	43.018	43.017	-23.2
	Acetol	$C_3H_6NO_3^+$	$[M+NO]^+$	104.034	104.032	-19.2
	Acetone	$C_3H_6NO_2^+$	$[M+NO]^+$	88.039	88.036	-34.1
	Glycerin	$C_3H_6NO_4^+$	$[M\text{-}2H+NO]^+$	120.029	120.027	-16.7
	Glycerin	$C_3H_7O_3^+$	$[M\text{-}H]^+$	91.039	91.036	-33.0
	Glycerin	$C_2H_5O_2^+$	$[M\text{-}H\text{-}CH_2O]^+$	61.028	61.025	-49.2
	Glycidol	$C_3H_6NO_4^+$	$[M\text{-}2H+H_2O+NO]^+$	120.029	120.024	-41.7
	Glycidol	$C_3H_6NO_3^+$	$[M+NO]^+$	104.034	104.033	-9.6
	Glycidol	$C_3H_7O_3^+$	$[M\text{-}H+H_2O]^+$	91.039	91.036	-33.0
	Glycidol	$C_3H_5O_2^+$	$[M\text{-}H]^+$	73.028	73.025	-41.1

Reagent ion	Compound	Ion formula	Mechanism	Theoretical mass	Experimental mass	Mass accuracy [ppm]
	Propylene glycol	$C_3H_6NO_3^+$	$[M-2H+NO]^+$	104.034	104.031	-28.8
	Propylene glycol	$C_3H_7O_2^+$	$[M-H]^+$	75.044	75.040	-53.3
NH_4^+	Acetaldehyde	-	-	-	-	-
	Acetol	$C_3H_{10}NO_2^+$	$[M+NH_4]^+$	92.071	92.063	-86.9
	Acetone	$C_3H_{10}NO^+$	$[M+NH_4]^+$	76.076	76.084	105.2
	Glycidol	$C_3H_{10}NO_2^+$	$[M+NH_4]^+$	92.071	92.063	-86.9
	Glycerin	$C_3H_{12}NO_3^+$	$[M+NH_4]^+$	110.081	110.078	-27.3
	Propylene glycol	$C_3H_{12}NO_2^+$	$[M+NH_4]^+$	94.086	94.077	-95.7

Table S3. Ratio between concentration of glycerin to sum of concentrations of acetaldehyde and glycidol for Mixture 1.

			**Acetaldehyde**							**Acetaldehyde**							**Acetaldehyde**					
		ppbV	25.62	19.21	12.81	6.40	2.56	1.28		1.28	2.56	6.40	12.81	19.21	25.62		25.62	19.21	12.81	6.40	2.56	1.28
		cps	52863	37986	23128	8125	3029	804		804	3029	8125	23128	37986	52863		52863	37986	23128	8125	3029	804
			**Glycidol**							**Glycidol**							**Glycidol**					
		ppbV	6.07	4.55	3.04	1.52	0.61	0.30		6.07	4.55	3.04	1.52	0.61	0.30		0.30	0.61	1.52	3.04	4.55	6.07
	ppbV	cps	4643	3463	2309	1174	451	201		4643	3463	2309	1174	451	201		201	451	1174	2309	3463	4643
**Glycerin**	200.2	132024	6.3	8.4	12.6	25.3	63.2	126.4		27.2	28.1	21.2	14.0	10.1	7.7		7.7	10.1	14.0	21.2	28.1	27.2
**Glycerin**	150.2	96489	4.7	6.3	9.5	19.0	47.4	94.8		20.4	21.1	15.9	10.5	7.6	5.8		5.8	7.6	10.5	15.9	21.1	20.4
**Glycerin**	100.1	60770	3.2	4.2	6.3	12.6	31.6	63.2		13.6	14.1	10.6	7.0	5.1	3.9		3.9	5.1	7.0	10.6	14.1	13.6
**Glycerin**	50.1	27059	1.6	2.1	3.2	6.3	15.8	31.6		6.8	7.0	5.3	3.5	2.5	1.9		1.9	2.5	3.5	5.3	7.0	6.8
**Glycerin**	20.0	8133	0.6	0.8	1.3	2.5	6.3	12.6		2.7	2.8	2.1	1.4	1.0	0.8		0.8	1.0	1.4	2.1	2.8	2.7
**Glycerin**	10.0	6396	0.3	0.4	0.6	1.3	3.2	6.3		1.4	1.4	1.1	0.7	0.5	0.4		0.4	0.5	0.7	1.1	1.4	1.4

Abbreviations: cps (counts per sec corrected for transmission and multiplier), ppbV (parts per billion by volume).

Table S4. Ratio between concentration of acetaldehyde to sum of concentrations of glycerin and glycidol for Mixture 1.

			**Acetaldehyde**							**Acetaldehyde**							**Acetaldehyde**					
		ppbV	25.62	19.21	12.81	6.40	2.56	1.28		1.28	2.56	6.40	12.81	19.21	25.62		25.62	19.21	12.81	6.40	2.56	1.28
		cps	52863	37986	23128	8125	3029	804		804	3029	8125	23128	37986	52863		52863	37986	23128	8125	3029	804
			**Glycidol**							**Glycidol**							**Glycidol**					
		ppbV	6.07	4.55	3.04	1.52	0.61	0.30		6.07	4.55	3.04	1.52	0.61	0.30		0.30	0.61	1.52	3.04	4.55	6.07
	ppbV	cps	4643	3463	2309	1174	451	201		4643	3463	2309	1174	451	201		201	451	1174	2309	3463	4643
**Glycerin**	200.2	132024	0.1	0.1	0.1	0.03	0.01	0.01		0.01	0.01	0.03	0.06	0.10	0.13		0.1	0.1	0.1	0.0	0.01	0.01
**Glycerin**	150.2	96489	0.2	0.1	0.1	0.04	0.02	0.01		0.01	0.02	0.04	0.08	0.13	0.17		0.2	0.1	0.1	0.0	0.02	0.01
**Glycerin**	100.1	60770	0.2	0.2	0.1	0.06	0.03	0.01		0.01	0.02	0.06	0.13	0.19	0.26		0.3	0.2	0.1	0.1	0.02	0.01
**Glycerin**	50.1	27059	0.5	0.4	0.2	0.12	0.05	0.03		0.02	0.05	0.12	0.25	0.38	0.51		0.5	0.4	0.2	0.1	0.05	0.02
**Glycerin**	20.0	8133	1.0	0.8	0.6	0.3	0.1	0.1		0.05	0.10	0.28	0.59	0.93	1.26		1.3	0.9	0.6	0.3	0.10	0.05
**Glycerin**	10.0	6396	1.6	1.3	1.0	0.6	0.2	0.1		0.08	0.18	0.5	1.1	1.8	2.5		2.5	1.8	1.1	0.5	0.18	0.08

Abbreviations: cps (counts per sec corrected for transmission and multiplier), ppbV (parts per billion by volume).

Table S5. Ratio between concentration of glycidol to sum of concentrations of glycerin and acetaldehyde for Mixture 1.

			**Acetaldehyde**							**Acetaldehyde**							**Acetaldehyde**					
		ppbV	25.62	19.21	12.81	6.40	2.56	1.28		1.28	2.56	6.40	12.81	19.21	25.62		25.62	19.21	12.81	6.40	2.56	1.28
		cps	52863	37986	23128	8125	3029	804		804	3029	8125	23128	37986	52863		52863	37986	23128	8125	3029	804
			**Glycidol**							**Glycidol**							**Glycidol**					
		ppbV	6.07	4.55	3.04	1.52	0.61	0.30		6.07	4.55	3.04	1.52	0.61	0.30		0.30	0.61	1.52	3.04	4.55	6.07
	ppbV	cps	4643	3463	2309	1174	451	201		4643	3463	2309	1174	451	201		201	451	1174	2309	3463	4643
**Glycerin**	200.2	132024	0.03	0.02	0.01	0.01	0.00	0.00		0.0	0.0	0.01	0.01	0.00	0.00		0.00	0.00	0.01	0.01	0.02	0.03
**Glycerin**	150.2	96489	0.03	0.03	0.02	0.01	0.00	0.00		0.0	0.0	0.02	0.01	0.00	0.00		0.00	0.00	0.01	0.02	0.03	0.04
**Glycerin**	100.1	60770	0.05	0.04	0.03	0.01	0.01	0.00		0.1	0.0	0.03	0.01	0.01	0.00		0.00	0.01	0.01	0.03	0.04	0.06
**Glycerin**	50.1	27059	0.08	0.07	0.05	0.03	0.01	0.01		0.1	0.1	0.05	0.02	0.01	0.00		0.00	0.01	0.02	0.05	0.09	0.12
**Glycerin**	20.0	8133	0.13	0.12	0.09	0.06	0.03	0.01		0.3	0.2	0.11	0.05	0.02	0.01		0.01	0.02	0.05	0.11	0.20	0.29
**Glycerin**	10.0	6396	0.17	0.16	0.13	0.09	0.05	0.03		0.5	0.4	0.18	0.07	0.02	0.01		0.01	0.02	0.07	0.18	0.36	0.54

Abbreviations: cps (counts per sec corrected for transmission and multiplier), ppbV (parts per billion by volume).

Table S6. Ratio between concentration of glycerin to sum of concentrations of acetaldehyde and glycidol for Mixture 2.

			**Acetaldehyde**							**Acetaldehyde**							**Acetaldehyde**					
		ppbV	32.34	24.26	16.17	8.09	3.23	1.62		1.62	3.23	8.09	16.2	24.3	32.3		32.34	24.26	16.17	8.09	3.23	1.62
		cps	82469	61409	36575	13283	5694	1560		1560	5694	13283	36575	61409	82469		82469	61409	36575	13283	5694	1560
			**Glycidol**							**Glycidol**							**Glycidol**					
		ppbV	6.75	5.06	3.37	1.69	0.67	0.34		6.75	5.06	3.37	1.69	0.67	0.34		0.34	0.67	1.69	3.37	5.06	6.75
	ppbV	cps	8923	7208	5362	3556	1660	731		8923	7208	5362	3556	1660	731		731	1660	3556	5362	7208	8923
**Glycerin**	208.6	133092	5.3	7.1	10.7	21.3	53.4	106.7		24.9	25.1	18.2	11.7	8.4	6.4		6.4	8.4	11.7	18.2	25.1	24.9
**Glycerin**	156.4	99917	4.0	5.3	8.0	16.0	40.0	80.0		18.7	18.9	13.7	8.8	6.3	4.8		4.8	6.3	8.8	13.7	18.9	18.7
**Glycerin**	104.3	60963	2.7	3.6	5.3	10.7	26.7	53.4		12.5	12.6	9.1	5.8	4.2	3.2		3.2	4.2	5.8	9.1	12.6	12.5
**Glycerin**	52.1	29096	1.3	1.8	2.7	5.3	13.3	26.7		6.2	6.3	4.6	2.9	2.1	1.6		1.6	2.1	2.9	4.6	6.3	6.2
**Glycerin**	20.9	10895	0.5	0.7	1.1	2.1	5.3	10.7		2.5	2.5	1.8	1.2	0.8	0.6		0.6	0.8	1.2	1.8	2.5	2.5
**Glycerin**	10.4	6633	0.3	0.4	0.5	1.1	2.7	5.3		1.2	1.3	0.9	0.6	0.4	0.3		0.3	0.4	0.6	0.9	1.3	1.2

Abbreviations: cps (counts per sec corrected for transmission and multiplier), ppbV (parts per billion by volume).

Table S7. Ratio between concentration of acetaldehyde to sum of concentrations of glycerin and glycidol for Mixture 2.

			**Acetaldehyde**						**Acetaldehyde**						**Acetaldehyde**					
		ppbV	32.34	24.26	16.17	8.09	3.23	1.62	1.62	3.23	8.09	16.2	24.3	32.3	32.34	24.26	16.17	8.09	3.23	1.62
		cps	82469	61409	36575	13283	5694	1560	1560	5694	13283	36575	61409	82469	82469	61409	36575	13283	5694	1560
			**Glycidol**						**Glycidol**						**Glycidol**					
		ppbV	6.75	5.06	3.37	1.69	0.67	0.34	6.75	5.06	3.37	1.69	0.67	0.34	0.34	0.67	1.69	3.37	5.06	6.75
	ppbV	cps	8923	7208	5362	3556	1660	731	8923	7208	5362	3556	1660	731	731	1660	3556	5362	7208	8923
**Glycerin**	208.6	133092	0.2	0.1	0.1	0.04	0.02	0.01	0.01	0.02	0.04	0.08	0.12	0.15	0.2	0.1	0.1	0.0	0.02	0.01
**Glycerin**	156.4	99917	0.2	0.2	0.1	0.05	0.02	0.01	0.01	0.02	0.05	0.10	0.15	0.21	0.2	0.2	0.1	0.1	0.02	0.01
**Glycerin**	104.3	60963	0.3	0.2	0.2	0.08	0.03	0.02	0.01	0.03	0.08	0.15	0.23	0.31	0.3	0.2	0.2	0.1	0.03	0.01
**Glycerin**	52.1	29096	0.5	0.4	0.3	0.15	0.06	0.03	0.03	0.06	0.15	0.30	0.46	0.62	0.6	0.5	0.3	0.1	0.06	0.03
**Glycerin**	20.9	10895	1.2	0.9	0.7	0.4	0.2	0.1	0.06	0.12	0.33	0.72	1.13	1.53	1.5	1.1	0.7	0.3	0.12	0.06
**Glycerin**	10.4	6633	1.9	1.6	1.2	0.7	0.3	0.2	0.09	0.21	0.6	1.3	2	3	3.0	2.2	1.3	0.6	0.21	0.09

Abbreviations: cps (counts per sec corrected for transmission and multiplier), ppbV (parts per billion by volume).

Table S8. Ratio between concentration of glycidol to sum of concentrations of glycerin and acetaldehyde for Mixture 2.

			**Acetaldehyde**						**Acetaldehyde**						**Acetaldehyde**					
		ppbV	32.34	24.26	16.17	8.09	3.23	1.62	1.62	3.23	8.09	16.2	24.3	32.3	32.34	24.26	16.17	8.09	3.23	1.62
		cps	82469	61409	36575	13283	5694	1560	1560	5694	13283	36575	61409	82469	82469	61409	36575	13283	5694	1560
			**Glycidol**						**Glycidol**						**Glycidol**					
		ppbV	6.75	5.06	3.37	1.69	0.67	0.34	6.75	5.06	3.37	1.69	0.67	0.34	0.34	0.67	1.69	3.37	5.06	6.75
	ppbV	cps	8923	7208	5362	3556	1660	731	8923	7208	5362	3556	1660	731	731	1660	3556	5362	7208	8923
**Glycerin**	208.6	133092	0.03	0.02	0.02	0.01	0.00	0.00	0.0	0.0	0.02	0.01	0.00	0.00	0.00	0.00	0.01	0.02	0.02	0.03
**Glycerin**	156.4	99917	0.04	0.03	0.02	0.01	0.00	0.00	0.0	0.0	0.02	0.01	0.00	0.00	0.00	0.00	0.01	0.02	0.03	0.04
**Glycerin**	104.3	60963	0.05	0.04	0.03	0.02	0.01	0.00	0.1	0.0	0.03	0.01	0.01	0.00	0.00	0.01	0.01	0.03	0.05	0.06
**Glycerin**	52.1	29096	0.08	0.07	0.05	0.03	0.01	0.01	0.1	0.1	0.06	0.02	0.01	0.00	0.00	0.01	0.02	0.06	0.09	0.13
**Glycerin**	20.9	10895	0.13	0.11	0.09	0.06	0.03	0.02	0.3	0.2	0.12	0.05	0.01	0.01	0.01	0.01	0.05	0.12	0.21	0.30
**Glycerin**	10.4	6633	0.16	0.15	0.13	0.09	0.05	0.03	0.6	0.4	0.18	0.06	0.02	0.01	0.01	0.02	0.06	0.18	0.37	0.56

Abbreviations: cps (counts per sec corrected for transmission and multiplier), ppbV (parts per billion by volume).

Table S9. Ratio between concentration of glycerin to sum of concentrations of acetol and glycidol for Mixture 3.

			**Acetol**						**Acetol**						**Acetol**					
		ppbV	56.45	42.34	28.23	14.11	5.65	2.82	2.82	5.65	14.11	28.23	42.34	56.45	56.45	42.34	28.23	14.11	5.65	2.82
		cps	13558	9908	6468	3205	1223	551	551	1223	3205	6468	9908	13558	13558	9908	6468	3205	1223	551
			**Glycidol**						**Glycidol**						**Glycidol**					
		ppbV	6.87	5.15	3.43	1.72	0.69	0.34	6.87	5.15	3.43	1.72	0.69	0.34	0.34	0.69	1.72	3.43	5.15	6.87
	ppbV	cps	1675	1229	794	385	154	62	1675	1229	794	385	154	62	62	154	385	794	1229	1675
**Glycerin**	207.1	102922	3.3	4.4	6.5	13.1	32.7	65.4	21.4	19.2	11.8	6.9	4.8	3.6	3.6	4.8	6.9	11.8	19.2	21.4
**Glycerin**	155.3	74258	2.5	3.3	4.9	9.8	24.5	49.1	16.0	14.4	8.9	5.2	3.6	2.7	2.7	3.6	5.2	8.9	14.4	16.0
**Glycerin**	103.6	46388	1.6	2.2	3.3	6.5	16.4	32.7	10.7	9.6	5.9	3.5	2.4	1.8	1.8	2.4	3.5	5.9	9.6	10.7
**Glycerin**	51.8	21375	0.8	1.1	1.6	3.3	8.2	16.4	5.3	4.8	3.0	1.7	1.2	0.9	0.9	1.2	1.7	3.0	4.8	5.3
**Glycerin**	20.7	7845	0.3	0.4	0.7	1.3	3.3	6.5	2.1	1.9	1.2	0.7	0.5	0.4	0.4	0.5	0.7	1.2	1.9	2.1
**Glycerin**	10.4	4872	0.2	0.2	0.3	0.7	1.6	3.3	1.1	1.0	0.6	0.3	0.2	0.2	0.2	0.2	0.3	0.6	1.0	1.1

Abbreviations: cps (counts per sec corrected for transmission and multiplier), ppbV (parts per billion by volume).

Table S10. Ratio between concentration of acetol to sum of concentrations of glycerin and glycidol for Mixture 3.

			**Acetol**						**Acetol**						**Acetol**					
		ppbV	56.45	42.34	28.23	14.11	5.65	2.82	2.82	5.65	14.11	28.23	42.34	56.45	56.45	42.34	28.23	14.11	5.65	2.82
		cps	13558	9908	6468	3205	1223	551	551	1223	3205	6468	9908	13558	13558	9908	6468	3205	1223	551
			**Glycidol**						**Glycidol**						**Glycidol**					
		ppbV	6.87	5.15	3.43	1.72	0.69	0.34	6.87	5.15	3.43	1.72	0.69	0.34	0.34	0.69	1.72	3.43	5.15	6.87
	ppbV	cps	1675	1229	794	385	154	62	1675	1229	794	385	154	62	62	154	385	794	1229	1675
**Glycerin**	207.1	102922	0.3	0.2	0.1	0.1	0.0	0.0	0.0	0.0	0.1	0.1	0.2	0.3	0.3	0.2	0.1	0.1	0.0	0.0
**Glycerin**	155.3	74258	0.3	0.3	0.2	0.1	0.0	0.0	0.0	0.0	0.1	0.2	0.3	0.4	0.4	0.3	0.2	0.1	0.0	0.0
**Glycerin**	103.6	46388	0.5	0.4	0.3	0.1	0.1	0.0	0.0	0.1	0.1	0.3	0.4	0.5	0.5	0.4	0.3	0.1	0.1	0.0
**Glycerin**	51.8	21375	1.0	0.7	0.5	0.3	0.1	0.1	0.0	0.1	0.3	0.5	0.8	1.1	1.1	0.8	0.5	0.3	0.1	0.0
**Glycerin**	20.7	7845	2.0	1.6	1.2	0.6	0.3	0.1	0.1	0.2	0.6	1.3	2.0	2.7	2.7	2.0	1.3	0.6	0.2	0.1
**Glycerin**	10.4	4872	3.3	2.7	2.0	1.2	0.5	0.3	0.2	0.4	1.0	2.3	3.8	5.3	5.3	3.8	2.3	1.0	0.4	0.2

Abbreviations: cps (counts per sec corrected for transmission and multiplier), ppbV (parts per billion by volume).

Table S11. Ratio between concentration of glycidol to sum of concentrations of glycerin and acetol for Mixture 3.

			Acetol						Acetol						Acetol					
		ppbV	56.45	42.34	28.23	14.11	5.65	2.82	2.82	5.65	14.11	28.23	42.34	56.45	56.45	42.34	28.23	14.11	5.65	2.82
		cps	13558	9908	6468	3205	1223	551	551	1223	3205	6468	9908	13,558	13558	9908	6468	3205	1223	551
			**Glycidol**						**Glycidol**						**Glycidol**					
		ppbV	6.87	5.15	3.43	1.72	0.69	0.34	6.87	5.15	3.43	1.72	0.69	0.34	0.34	0.69	1.72	3.43	5.15	6.87
	ppbV	cps	1675	1229	794	385	154	62	1675	1229	794	385	154	62	62	154	385	794	1229	1675
**Glycerin**	207.1	102922	0.03	0.02	0.01	0.01	0.00	0.00	0.03	0.02	0.02	0.01	0.00	0.00	0.00	0.00	0.01	0.02	0.02	0.03
**Glycerin**	155.3	74258	0.03	0.03	0.02	0.01	0.00	0.00	0.04	0.03	0.02	0.01	0.00	0.00	0.00	0.00	0.01	0.02	0.03	0.04
**Glycerin**	103.6	46388	0.04	0.04	0.03	0.01	0.01	0.00	0.06	0.05	0.03	0.01	0.00	0.00	0.00	0.00	0.01	0.03	0.05	0.06
**Glycerin**	51.8	21375	0.06	0.05	0.04	0.03	0.01	0.01	0.13	0.09	0.05	0.02	0.01	0.00	0.00	0.01	0.02	0.05	0.09	0.13
**Glycerin**	20.7	7845	0.09	0.08	0.07	0.05	0.03	0.01	0.29	0.20	0.10	0.04	0.01	0.00	0.00	0.01	0.04	0.10	0.20	0.29
**Glycerin**	10.4	4872	0.10	0.10	0.09	0.07	0.04	0.03	0.52	0.32	0.14	0.04	0.01	0.01	0.01	0.01	0.04	0.14	0.32	0.52

Abbreviations: cps (counts per sec corrected for transmission and multiplier), ppbV (parts per billion by volume).

Table S12. Influence on Ion 1. Percent glycerin fragment $[^{13}C_2D_3H_2O]^+$ vs. sum of glycerin fragment $[^{13}C_2D_3H_2O]^+$, acetaldehyde $[C_2H_5O]^+$, and glycidol fragment $[C_2D_3H_2O]^+$ in Mixture 1.

			Acetaldehyde						Acetaldehyde						Acetaldehyde					
		ppbV	25.62	19.21	12.81	6.40	2.56	1.28	1.28	2.56	6.40	12.81	19.21	25.62	25.62	19.21	12.81	6.40	2.56	1.28
		cps	52863	37986	23128	8125	3029	804	804	3029	8125	23128	37986	52863	52863	37986	23128	8125	3029	804
			**Glycidol**						**Glycidol**						**Glycidol**					
		ppbV	6.07	4.55	3.04	1.52	0.61	0.30	6.07	4.55	3.04	1.52	0.61	0.30	0.30	0.61	1.52	3.04	4.55	6.07
	ppbV	cps	4643	3463	2309	1174	451	201	4643	3463	2309	1174	451	201	201	451	1174	2309	3463	4643
**Glycerin**	200.2	132024	70%	76%	84%	93%	97%	99%	96%	95%	93%	84%	77%	71%	71%	77%	84%	93%	95%	96%
**Glycerin**	150.2	96489	63%	70%	79%	91%	97%	99%	95%	94%	90%	80%	72%	65%	65%	72%	80%	90%	94%	95%
**Glycerin**	100.1	60770	51%	59%	70%	87%	95%	98%	92%	90%	85%	71%	61%	53%	53%	61%	71%	85%	90%	92%
**Glycerin**	50.1	27059	32%	39%	52%	74%	89%	96%	83%	81%	72%	53%	41%	34%	34%	41%	53%	72%	81%	83%
**Glycerin**	20.0	8133	12%	16%	24%	47%	70%	89%	60%	56%	44%	25%	17%	13%	13%	17%	25%	44%	56%	60%
**Glycerin**	10.0	6396	10%	13%	20%	41%	65%	86%	54%	50%	38%	21%	14%	11%	11%	14%	21%	38%	50%	54%

Abbreviations: cps (counts per sec corrected for transmission and multiplier), ppbV (parts per billion by volume).

Table S13. Influence on Ion 1. Percent glycerin fragment $[^{13}C_2D_3H_2O]^+$ vs. sum of glycerin fragment $[^{13}C_2D_3H_2O]^+$, acetaldehyde $[^{13}C_2H_5O]^+$, and glycidol fragment $[C_2H_5O]^+$ in Mixture 2.

			**Acetaldehyde**						**Acetaldehyde**						**Acetaldehyde**					
		ppbV	32.34	24.26	16.17	8.09	3.23	1.62	1.62	3.23	8.09	16.2	24.3	32.3	32.34	24.26	16.17	8.09	3.23	1.62
		cps	82469	61409	36575	13283	5694	1560	1560	5694	13283	36575	61409	82469	82469	61409	36575	13283	5694	1560
			**Glycidol**						**Glycidol**						**Glycidol**					
		ppbV	6.75	5.06	3.37	1.69	0.67	0.34	6.75	5.06	3.37	1.69	0.67	0.34	0.34	0.67	1.69	3.37	5.06	6.75
	ppbV	cps	8923	7208	5362	3556	1660	731	8923	7208	5362	3556	1660	731	731	1660	3556	5362	7208	8923
**Glycerin**	208.6	133092	59%	66%	76%	89%	95%	98%	93%	91%	88%	77%	68%	62%	62%	68%	77%	88%	91%	93%
**Glycerin**	156.5	99917	52%	59%	70%	86%	93%	98%	91%	89%	84%	71%	61%	55%	55%	61%	71%	84%	89%	91%
**Glycerin**	104.3	60963	40%	47%	59%	78%	89%	96%	85%	83%	77%	60%	49%	42%	42%	49%	60%	77%	83%	85%
**Glycerin**	52.1	29096	24%	30%	41%	63%	80%	93%	74%	69%	61%	42%	32%	26%	26%	32%	42%	61%	69%	74%
**Glycerin**	20.9	10895	11%	14%	21%	39%	60%	83%	51%	46%	37%	21%	15%	12%	12%	15%	21%	37%	46%	51%
**Glycerin**	10.4	6633	7%	9%	14%	28%	47%	74%	39%	34%	26%	14%	10%	7%	7%	10%	14%	26%	34%	39%

Abbreviations: cps (counts per sec corrected for transmission and multiplier), ppbV (parts per billion by volume).

Table S14. Influence on Ion 1. Percent acetaldehyde $[C_2H_5O]^+$ vs. sum of glycerin fragment $[^{13}C_2D_3H_2O]^+$, acetaldehyde $[C_2H_5O]^+$, and glycidol fragment $[C_2D_3H_2O]^+$ in Mixture 1.

			**Acetaldehyde**						**Acetaldehyde**						**Acetaldehyde**					
		ppbV	25.62	19.21	12.81	6.40	2.56	1.28	1.28	2.56	6.40	12.81	19.21	25.62	25.62	19.21	12.81	6.40	2.56	1.28
		cps	52863	37986	23128	8125	3029	804	804	3029	8125	23128	37986	52863	52863	37986	23128	8125	3029	804
			**Glycidol**						**Glycidol**						**Glycidol**					
		ppbV	6.07	4.55	3.04	1.52	0.61	0.30	6.07	4.55	3.04	1.52	0.61	0.30	0.30	0.61	1.52	3.04	4.55	6.07
	ppbV	cps	4643	3463	2309	1174	451	201	4643	3463	2309	1174	451	201	201	451	1174	2309	3463	4643
**Glycerin**	200.2	132024	28%	22%	15%	6%	2%	1%	1%	2%	6%	15%	22%	29%	29%	22%	15%	6%	2%	1%
**Glycerin**	150.2	96489	34%	28%	19%	8%	3%	1%	1%	3%	8%	19%	28%	35%	35%	28%	19%	8%	3%	1%
**Glycerin**	100.1	60770	45%	37%	27%	12%	5%	1%	1%	5%	11%	27%	38%	46%	46%	38%	27%	11%	5%	1%
**Glycerin**	50.1	27059	63%	55%	44%	22%	10%	3%	2%	9%	22%	45%	58%	66%	66%	58%	45%	22%	9%	2%
**Glycerin**	20.0	8133	81%	77%	69%	47%	26%	9%	6%	21%	44%	71%	82%	86%	86%	82%	71%	44%	21%	6%
**Glycerin**	10.0	6396	83%	79%	73%	52%	31%	11%	7%	24%	48%	75%	85%	89%	89%	85%	75%	48%	24%	7%

Abbreviations: cps (counts per sec corrected for transmission and multiplier), ppbV (parts per billion by volume).

Table S15. Influence on Ion 1. Percent acetaldehyde $[^{13}C_2H_5O]^+$ vs. sum of glycerin fragment $[^{13}C_2D_3H_2O]^+$, acetaldehyde $[^{13}C_2H_5O]^+$, and glycidol fragment $[C_2H_5O]^+$ in Mixture 2.

			**Acetaldehyde**						**Acetaldehyde**						**Acetaldehyde**					
		ppbV	32.34	24.26	16.17	8.09	3.23	1.62	1.62	3.23	8.09	16.2	24.3	32.3	32.34	24.26	16.17	8.09	3.23	1.62
		cps	82469	61409	36575	13283	5694	1560	1560	5694	13283	36575	61409	82469	82469	61409	36575	13283	5694	1560
			**Glycidol**						**Glycidol**						**Glycidol**					
		ppbV	6.75	5.06	3.37	1.69	0.67	0.34	6.75	5.06	3.37	1.69	0.67	0.34	0.34	0.67	1.69	3.37	5.06	6.75
	ppbV	cps	8923	7208	5362	3556	1660	731	8923	7208	5362	3556	1660	731	731	1660	3556	5362	7208	8923
**Glycerin**	208.6	133092	37%	30%	21%	9%	4%	1%	1%	4%	9%	21%	31%	38%	38%	31%	21%	9%	4%	1%
**Glycerin**	156.5	99917	43%	36%	26%	11%	5%	2%	1%	5%	11%	26%	38%	45%	45%	38%	26%	11%	5%	1%
**Glycerin**	104.3	60963	54%	47%	36%	17%	8%	2%	2%	8%	17%	36%	50%	57%	57%	50%	36%	17%	8%	2%
**Glycerin**	52.1	29096	68%	63%	51%	29%	16%	5%	4%	14%	28%	53%	67%	73%	73%	67%	53%	28%	14%	4%
**Glycerin**	20.9	10895	81%	77%	69%	48%	31%	12%	7%	24%	45%	72%	83%	88%	88%	83%	72%	45%	24%	7%
**Glycerin**	10.4	6633	84%	82%	75%	57%	41%	17%	9%	29%	53%	78%	88%	92%	92%	88%	78%	53%	29%	9%

Abbreviations: cps (counts per sec corrected for transmission and multiplier), ppbV (parts per billion by volume).

Table S16. Influence on Ion 1. Percent glycidol fragment $[C_2D_3H_2O]^+$ vs. sum of glycerin fragment $[^{13}C_2D_3H_2O]^+$, acetaldehyde $[C_2H_5O]^+$, and glycidol fragment $[C_2D_3H_2O]^+$ in Mixture 1.

			**Acetaldehyde**						**Acetaldehyde**						**Acetaldehyde**					
		ppbV	25.62	19.21	12.81	6.40	2.56	1.28	1.28	2.56	6.40	12.81	19.21	25.62	25.62	19.21	12.81	6.40	2.56	1.28
		cps	52863	37986	23128	8125	3029	804	804	3029	8125	23128	37986	52863	52863	37986	23128	8125	3029	804
			**Glycidol**						**Glycidol**						**Glycidol**					
		ppbV	6.07	4.55	3.04	1.52	0.61	0.30	0.30	0.61	1.52	3.04	4.55	6.07	0.30	0.61	1.52	3.04	4.55	6.07
	ppbV	cps	4643	3463	2309	1174	451	201	201	451	1174	2309	3463	4643	201	451	1174	2309	3463	4643
**Glycerin**	200.2	132024	2.4%	2.0%	1.5%	0.8%	0.3%	0.2%	0.2%	0.3%	0.8%	1.5%	2.0%	2.4%	0.1%	0.3%	0.8%	1.6%	2.5%	3.4%
**Glycerin**	150.2	96489	3.0%	2.5%	1.9%	1.1%	0.5%	0.2%	0.2%	0.5%	1.1%	1.9%	2.5%	3.0%	0.1%	0.3%	1.0%	2.2%	3.4%	4.6%
**Glycerin**	100.1	60770	3.9%	3.4%	2.7%	1.7%	0.7%	0.3%	0.3%	0.7%	1.7%	2.7%	3.4%	3.9%	0.2%	0.5%	1.4%	3.2%	5.1%	7.0%
**Glycerin**	50.1	27059	5.5%	5.1%	4.4%	3.2%	1.5%	0.7%	0.7%	1.5%	3.2%	4.4%	5.1%	5.5%	0.3%	0.7%	2.3%	6.2%	10.3%	14.3%
**Glycerin**	20.0	8133	7.1%	7.0%	6.9%	6.7%	3.9%	2.2%	2.2%	3.9%	6.7%	6.9%	7.0%	7.1%	0.3%	1.0%	3.6%	12.4%	23.7%	34.2%
**Glycerin**	10.0	6396	7.3%	7.2%	7.3%	7.5%	4.6%	2.7%	2.7%	4.6%	7.5%	7.3%	7.2%	7.3%	0.3%	1.0%	3.8%	13.7%	26.9%	39.2%

Abbreviations: cps (counts per sec corrected for transmission and multiplier), ppbV (parts per billion by volume).

Table S17. Influence on Ion 1. Percent glycidol fragment $[C_2H_5O]^+$ vs. sum of glycerin fragment $[^{13}C_2D_3H_2O]^+$, acetaldehyde $[^{13}C_2H_5O]^+$, and glycidol fragment $[C_2H_5O]^+$ in Mixture 2.

			**Acetaldehyde**						**Acetaldehyde**						**Acetaldehyde**					
		ppbV	32.34	24.26	16.17	8.09	3.23	1.62	1.62	3.23	8.09	16.2	24.3	32.3	32.34	24.26	16.17	8.09	3.23	1.62
		cps	82469	61409	36575	13283	5694	1560	1560	5694	13283	36575	61409	82469	8246<br>9	61409	36575	13283	5694	1560
			**Glycidol**						**Glycidol**						**Glycidol**					
		ppbV	6.75	5.06	3.37	1.69	0.67	0.34	6.75	5.06	3.37	1.69	0.67	0.34	0.34	0.67	1.69	3.37	5.06	6.75
	ppbV	cps	8923	7208	5362	3556	1660	731	8923	7208	5362	3556	1660	731	731	1660	3556	5362	7208	8923
**Glycerin**	208.6	133092	4%	4%	3%	2%	1.2%	0.5%	6.2%	5%	4%	2%	1%	0%	0.3%	1%	2%	4%	5%	6%
**Glycerin**	156.5	99917	5%	4%	4%	3%	1.5%	0.7%	8.1%	6%	5%	3%	1%	0%	0.4%	1%	3%	5%	6%	8%
**Glycerin**	104.3	60963	6%	6%	5%	5%	2%	1%	12%	10%	7%	4%	1%	1%	0.5%	1%	4%	7%	10%	12%
**Glycerin**	52.1	29096	7%	7%	8%	8%	5%	2%	22%	17%	11%	5%	2%	1%	0.7%	2%	5%	11%	17%	23%
**Glycerin**	20.9	10895	9%	9%	10%	13%	9%	6%	42%	30%	18%	7%	2%	1%	0.8%	2%	7%	18%	30%	42%
**Glycerin**	10.4	6633	9%	10%	11%	15%	12%	8%	52%	37%	21%	8%	2%	1%	0.8%	2%	8%	21%	37%	52%

Abbreviations: cps (counts per sec corrected for transmission and multiplier), ppbV (parts per billion by volume).

Table S18. Influence on Ion 2. Percent glycerin fragment $[^{13}C_3D_5O]^+$ vs. sum of glycerin fragment $[^{13}C_3D_5O]^+$, acetol fragment $[C_3H_5O]^+$, and glycidol fragment $[C_3D_5O]^+$ in Mixture 3.

			**Acetol**						**Acetol**						**Acetol**					
		ppbV	56.45	42.34	28.23	14.11	5.65	2.82	2.82	5.65	14.11	28.23	42.34	56.45	56.45	42.34	28.23	14.11	5.65	2.82
		cps	13558	9908	6468	3205	1223	551	551	1223	3205	6468	9908	13558	13558	9908	6468	3205	1223	551
			**Glycidol**						**Glycidol**						**Glycidol**					
		ppbV	6.87	5.15	3.43	1.72	0.69	0.34	6.87	5.15	3.43	1.72	0.69	0.34	0.34	0.69	1.72	3.43	5.15	6.87
	ppbV	cps	1675	1229	794	385	154	62	1675	1229	794	385	154	62	62	154	385	794	1229	1675
**Glycerin**	207.1	102922	87%	90%	93%	97%	99%	99%	98%	98%	96%	94%	91%	88%	88%	91%	94%	96%	98%	98%
**Glycerin**	155.3	74258	83%	87%	91%	95%	98%	99%	97%	97%	95%	92%	88%	85%	85%	88%	92%	95%	97%	97%
**Glycerin**	103.6	46388	75%	81%	86%	93%	97%	99%	95%	95%	92%	87%	82%	77%	77%	82%	87%	92%	95%	95%
**Glycerin**	51.8	21375	58%	66%	75%	86%	94%	97%	91%	90%	84%	76%	68%	61%	61%	68%	76%	84%	90%	91%
**Glycerin**	20.7	7845	34%	41%	52%	69%	85%	93%	78%	76%	66%	53%	44%	37%	37%	44%	53%	66%	76%	78%
**Glycerin**	10.4	4872	24%	30%	40%	58%	78%	89%	69%	67%	55%	42%	33%	26%	26%	33%	42%	55%	67%	69%

Abbreviations: cps (counts per sec corrected for transmission and multiplier), ppbV (parts per billion by volume).

Table S19. Influence on Ion 2. Percent acetol fragment $[C_3H_5O]^+$ vs. sum of glycerin fragment $[^{13}C_3D_5O]^+$, acetol fragment $[C_3H_5O]^+$, and glycidol fragment $[C_3D_5O]^+$ in Mixture 3.

			**Acetol**						**Acetol**						**Acetol**					
		ppbV	56.45	42.34	28.23	14.11	5.65	2.82	2.82	5.65	14.11	28.23	42.34	56.45	56.45	42.34	28.23	14.11	5.65	2.82
		cps	13558	9908	6468	3205	1223	551	551	1223	3205	6468	9908	13558	13558	9908	6468	3205	1223	551
			**Glycidol**						**Glycidol**						**Glycidol**					
		ppbV	6.87	5.15	3.43	1.72	0.69	0.34	6.87	5.15	3.43	1.72	0.69	0.34	0.34	0.69	1.72	3.43	5.15	6.87
	ppbV	cps	1675	1229	794	385	154	62	1675	1229	794	385	154	62	62	154	385	794	1229	1675
**Glycerin**	207.1	102922	11%	9%	6%	3%	1%	1%	1%	1%	3%	6%	9%	12%	12%	9%	6%	3%	1%	1%
**Glycerin**	155.3	74258	15%	12%	8%	4%	2%	1%	1%	2%	4%	8%	12%	15%	15%	12%	8%	4%	2%	1%
**Glycerin**	103.6	46388	22%	17%	12%	6%	3%	1%	1%	3%	6%	12%	18%	23%	23%	18%	12%	6%	3%	1%
**Glycerin**	51.8	21375	37%	30%	23%	13%	5%	3%	2%	5%	13%	23%	32%	39%	39%	32%	23%	13%	5%	2%
**Glycerin**	20.7	7845	59%	52%	43%	28%	13%	7%	5%	12%	27%	44%	55%	63%	63%	55%	44%	27%	12%	5%
**Glycerin**	10.4	4872	67%	62%	53%	38%	20%	10%	8%	17%	36%	55%	66%	73%	73%	66%	55%	36%	17%	8%

Abbreviations: cps (counts per sec corrected for transmission and multiplier), ppbV (parts per billion by volume).

Table S20. Influence on Ion 2. Percent glycidol fragment $[C_3D_5O]^+$ vs. sum of glycerin fragment $[^{13}C_3D_5O]^+$, acetol fragment $[C_3H_5O]^+$, and gycidol fragment $[C_3D_5O]^+$ in Mixture 3.

			**Acetol**						**Acetol**						**Acetol**					
		ppbV	56.45	42.34	28.23	14.11	5.65	2.82	2.82	5.65	14.11	28.23	42.34	56.45	56.45	42.34	28.23	14.11	5.65	2.82
		cps	13558	9908	6468	3205	1223	551	551	1223	3205	6468	9908	13558	13558	9908	6468	3205	1223	551
			**Glycidol**						**Glycidol**						**Glycidol**					
		ppbV	6.87	5.15	3.43	1.72	0.69	0.34	6.87	5.15	3.43	1.72	0.69	0.34	0.34	0.69	1.72	3.43	5.15	6.87
	ppbV	cps	1675	1229	794	385	154	62	1675	1229	794	385	154	62	62	154	385	794	1229	1675
**Glycerin**	207.1	102922	1.4%	1.1%	0.7%	0.4%	0.1%	0.1%	1.6%	1.2%	0.7%	0.4%	0.1%	0.1%	0.1%	0.1%	0.4%	0.7%	1.2%	1.6%
**Glycerin**	155.3	74258	1.9%	1.4%	1.0%	0.5%	0.2%	0.1%	2.2%	1.6%	1.0%	0.5%	0.2%	0.1%	0.1%	0.2%	0.5%	1.0%	1.6%	2.2%
**Glycerin**	103.6	46388	2.7%	2.1%	1.5%	0.8%	0.3%	0.1%	3.4%	2.5%	1.6%	0.7%	0.3%	0.1%	0.1%	0.3%	0.7%	1.6%	2.5%	3.4%
**Glycerin**	51.8	21375	4.6%	3.8%	2.8%	1.5%	0.7%	0.3%	7.1%	5.2%	3.1%	1.4%	0.5%	0.2%	0.2%	0.5%	1.4%	3.1%	5.2%	7.1%
**Glycerin**	20.7	7845	7.3%	6.5%	5.3%	3.4%	1.7%	0.7%	17%	12%	6.7%	2.6%	0.9%	0.3%	0.3%	0.9%	2.6%	6.7%	12%	17%
**Glycerin**	10.4	4872	8.3%	7.7%	6.5%	4.5%	2.5%	1.1%	24%	17%	9.0%	3.3%	1.0%	0.3%	0.3%	1.0%	3.3%	9.0%	17%	24%

Abbreviations: cps (counts per sec corrected for transmission and multiplier), ppbV (parts per billion by volume).

Table S21. Influence on Ion 3. Percent glycerin fragment $[^{13}C_3D_5H_2O_2]^+$ vs. sum of glycerin fragment $[^{13}C_3D_5H_2O_2]^+$, acetol $[C_3H_7O_2]^+$, and glycidol $[C_3D_5H_2O_2]^+$ in Mixture 3.

			Acetol						Acetol						Acetol					
		ppbV	56.45	42.34	28.23	14.11	5.65	2.82	2.82	5.65	14.11	28.23	42.34	56.45	56.45	42.34	28.23	14.11	5.65	2.82
		cps	126494	94759	62503	30928	11405	5261	5261	11405	30928	62503	94759	126494	126494	94759	62503	30928	11405	5261
			Glycidol						Glycidol						Glycidol					
		ppbV	6.87	5.15	3.43	1.72	0.69	0.34	6.87	5.15	3.43	1.72	0.69	0.34	0.34	0.69	1.72	3.43	5.15	6.87
	ppbV	cps	942	678	440	204	79	41	942	678	440	204	79	41	41	79	204	440	678	942
**Glycerin**	207.1	134225	51%	58%	68%	81%	92%	96%	96%	92%	81%	68%	59%	51%	51%	59%	68%	81%	92%	96%
**Glycerin**	155.3	100293	44%	51%	61%	76%	90%	95%	94%	89%	76%	62%	51%	44%	44%	51%	62%	76%	89%	94%
**Glycerin**	103.6	63763	33%	40%	50%	67%	85%	92%	91%	84%	67%	50%	40%	34%	34%	40%	50%	67%	84%	91%
**Glycerin**	51.8	29755	19%	24%	32%	49%	72%	85%	83%	71%	49%	32%	24%	19%	19%	24%	32%	49%	71%	83%
**Glycerin**	20.7	10826	8%	10%	15%	26%	49%	67%	64%	47%	26%	15%	10%	8%	8%	10%	15%	26%	47%	64%
**Glycerin**	10.4	6712	5%	7%	10%	18%	37%	56%	52%	36%	18%	10%	7%	5%	5%	7%	10%	18%	36%	52%

Abbreviations: cps (counts per sec corrected for transmission and multiplier), ppbV (parts per billion by volume).

Table S22. Influence on Ion 3. Percent acetol $[C_3H_7O_2]^+$ vs. sum of glycerin fragment $[^{13}C_3D_5H_2O_2]^+$, acetol $[C_3H_7O_2]^+$, and glycidol $[C_3D_5H_2O_2]^+$ in Mixture 3.

			Acetol						Acetol						Acetol					
		ppbV	56.45	42.34	28.23	14.11	5.65	2.82	2.82	5.65	14.11	28.23	42.34	56.45	56.45	42.34	28.23	14.11	5.65	2.82
		cps	126494	94759	62503	30928	11405	5261	5261	11405	30928	62503	94759	126494	126494	94759	62503	30928	11405	5261
			Glycidol						Glycidol						Glycidol					
		ppbV	6.87	5.15	3.43	1.72	0.69	0.34	6.87	5.15	3.43	1.72	0.69	0.34	0.34	0.69	1.72	3.43	5.15	6.87
	ppbV	cps	942	678	440	204	79	41	942	678	440	204	79	41	41	79	204	440	678	942
**Glycerin**	207.1	134225	48%	41%	32%	19%	8%	4%	4%	8%	19%	32%	41%	49%	49%	41%	32%	19%	8%	4%
**Glycerin**	155.3	100293	56%	48%	38%	24%	10%	5%	5%	10%	23%	38%	49%	56%	56%	49%	38%	23%	10%	5%
**Glycerin**	103.6	63763	66%	60%	49%	33%	15%	8%	8%	15%	33%	49%	60%	66%	66%	60%	49%	33%	15%	8%
**Glycerin**	51.8	29755	80%	76%	67%	51%	28%	15%	15%	27%	51%	68%	76%	81%	81%	76%	68%	51%	27%	15%
**Glycerin**	20.7	10826	91%	89%	85%	74%	51%	33%	31%	50%	73%	85%	90%	92%	92%	90%	85%	73%	50%	31%
**Glycerin**	10.4	6712	94%	93%	90%	82%	63%	44%	41%	61%	81%	90%	93%	95%	95%	93%	90%	81%	61%	41%

Abbreviations: cps (counts per sec corrected for transmission and multiplier), ppbV (parts per billion by volume).

Table S23. Influence on Ion 3. Percent glycidol $[C_3D_5H_2O_2]^+$ vs. sum of glycerin fragment $[^{13}C_3D_5H_2O_2]^+$, acetol $[C_3H_7O_2]^+$, and glycidol $[C_3D_5H_2O_2]^+$ in Mixture 3.

			**Acetol**						**Acetol**						**Acetol**					
		ppbV	56.45	42.34	28.23	14.11	5.65	2.82	2.82	5.65	14.11	28.23	42.34	56.45	56.45	42.34	28.23	14.11	5.65	2.82
		cps	126494	94759	62503	30928	11405	5261	5261	11405	30928	62503	94759	126494	126494	94759	62503	30928	11405	5261
			**Glycidol**						**Glycidol**						**Glycidol**					
		ppbV	6.87	5.15	3.43	1.72	0.69	0.34	6.87	5.15	3.43	1.72	0.69	0.34	0.34	0.69	1.72	3.43	5.15	6.87
	ppbV	cps	942	678	440	204	79	41	942	678	440	204	79	41	41	79	204	440	678	942
**Glycerin**	207.1	134225	0%	0%	0%	0%	0%	0%	1%	0%	0%	0%	0%	0%	0%	0%	0%	0%	0%	1%
**Glycerin**	155.3	100293	0%	0%	0%	0%	0%	0%	1%	1%	0%	0%	0%	0%	0%	0%	0%	0%	1%	1%
**Glycerin**	103.6	63763	0%	0%	0%	0%	0%	0%	1%	1%	0%	0%	0%	0%	0%	0%	0%	0%	1%	1%
**Glycerin**	51.8	29755	1%	1%	0%	0%	0%	0%	3%	2%	1%	0%	0%	0%	0%	0%	0%	1%	2%	3%
**Glycerin**	20.7	10826	1%	1%	1%	0%	0%	0%	6%	3%	1%	0%	0%	0%	0%	0%	0%	1%	3%	6%
**Glycerin**	10.4	6712	1%	1%	1%	1%	0%	0%	7%	4%	1%	0%	0%	0%	0%	0%	0%	1%	4%	7%

Abbreviations: cps (counts per sec corrected for transmission and multiplier), ppbV (parts per billion by volume).

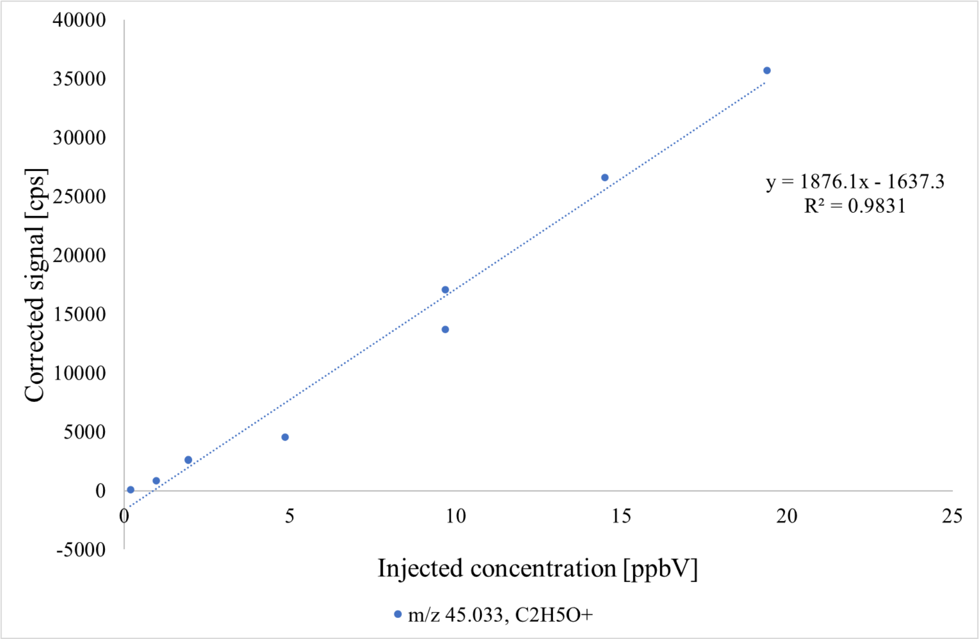

Figure S1. Calibration plot of response (cps) versus concentration (ppbV) for acetaldehyde in the H_3O^+ mode (E/N 69 Td). *NB: a second injection was performed mid-range for quality control purposes to verify the consistency of response.*

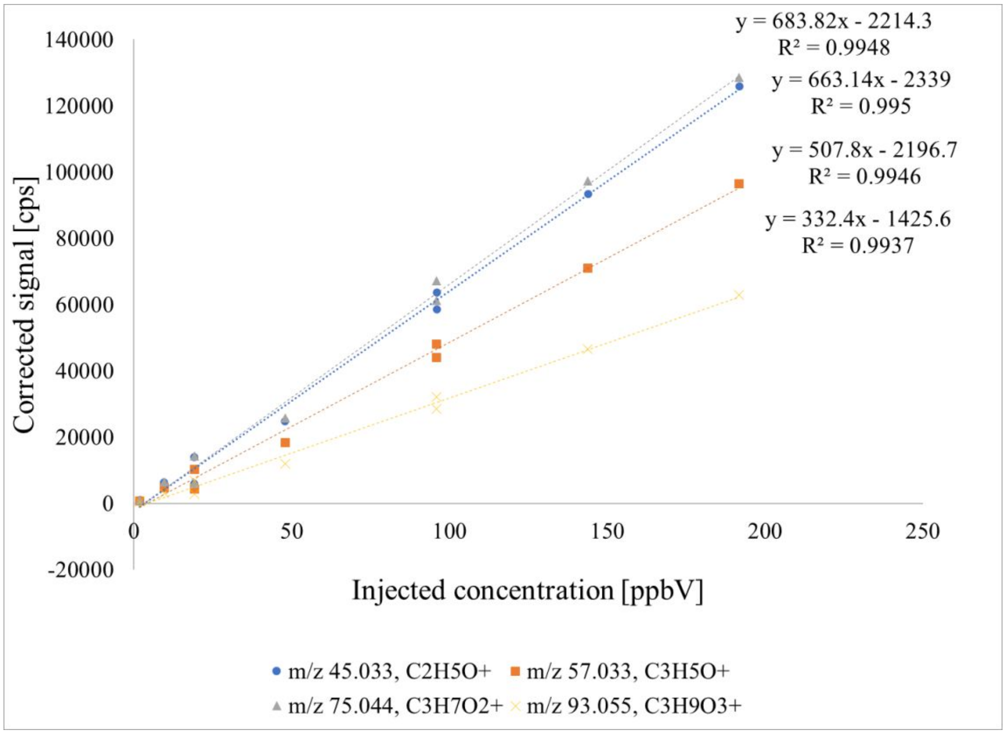

Figure S2. Calibration plot of response (cps) versus concentration (ppbV) for glycerin in H_3O^+ mode (E/N 69 Td). *NB: a second injection was performed mid-range for quality control purposes to verify the consistency of response.*

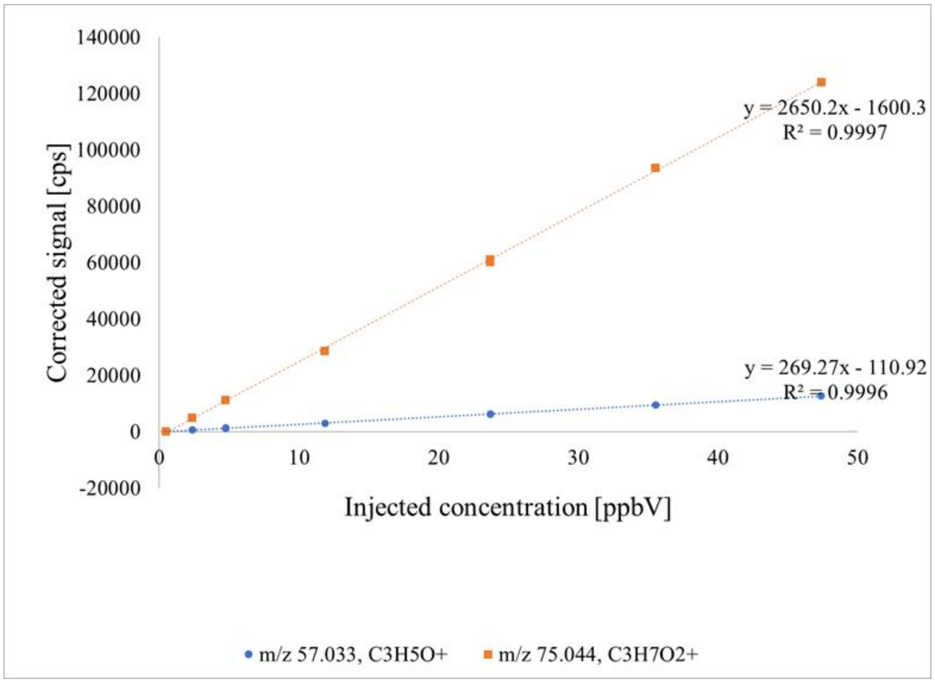

Figure S3. Calibration plot of response (cps) versus concentration (ppbV) for acetol in H_3O^+ mode (E/N 69 Td). *NB: a second injection was performed mid-range for quality control purposes to verify the consistency of response.*

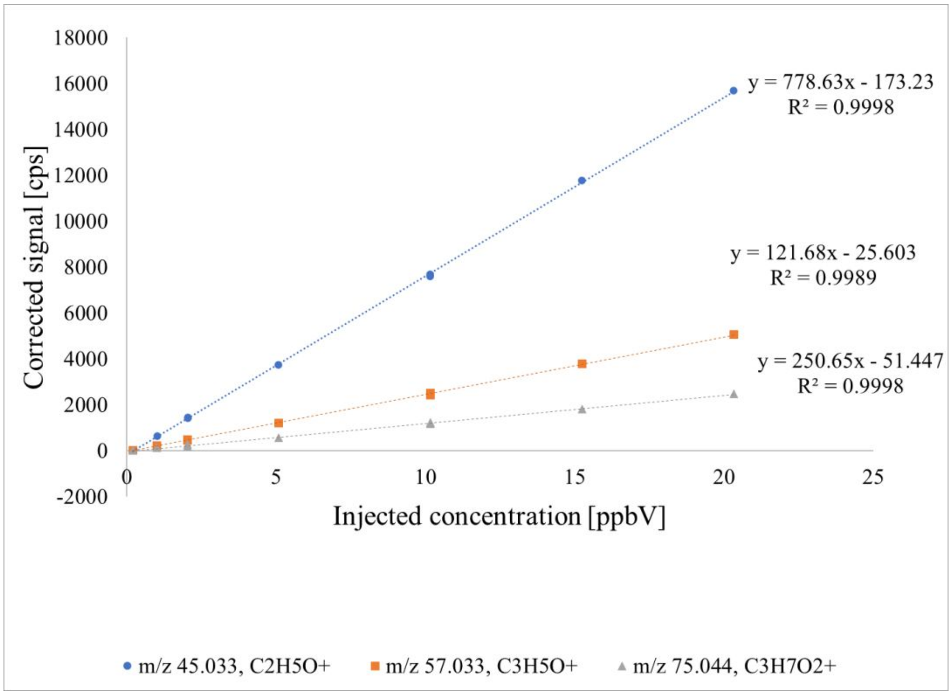

Figure S4. Calibration plot of response (cps) versus concentration (ppbV) for glycidol in H_3O^+ mode (E/N 69 Td). *NB: a second injection was performed mid-range for quality control purposes to verify the consistency of response.*

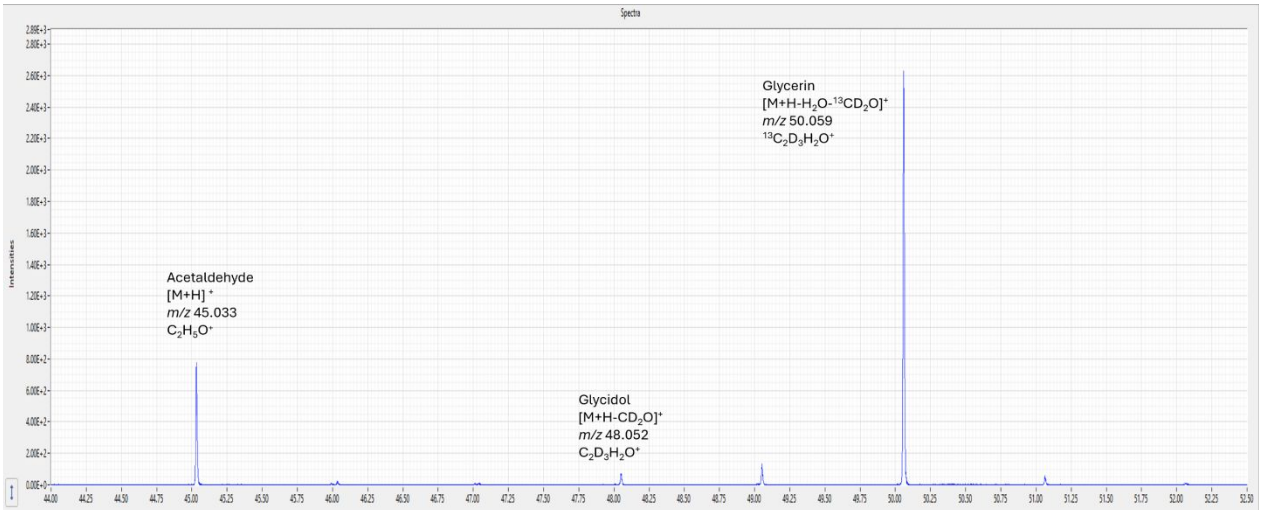

Figure S5. Mass spectrum of Ion 1 in Mixture 1: unlabeled acetaldehyde ($C_2H_5O^+$) and stable isotope-labeled fragments of glycerin ($^{13}C_2D_3H_2O^+$) and glycidol ($C_2D_3H_2O^+$).

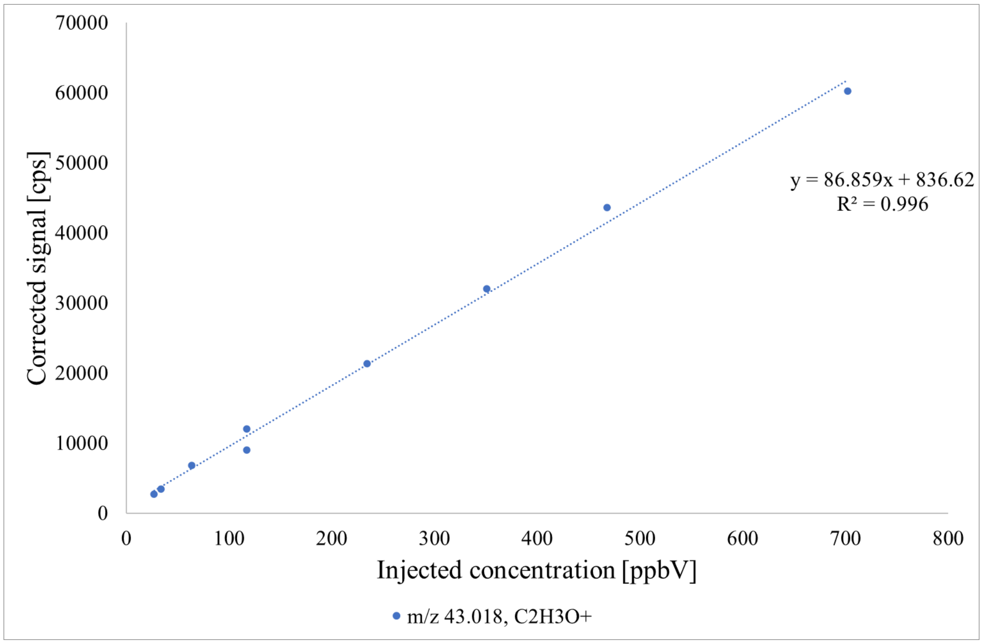

Figure S6. Calibration plot of response (cps) versus concentration (ppbV) for acetaldehyde in NO^+ mode (E/N 16 Td). *NB: a second injection was performed mid-range for quality control purposes to verify the consistency of response.*

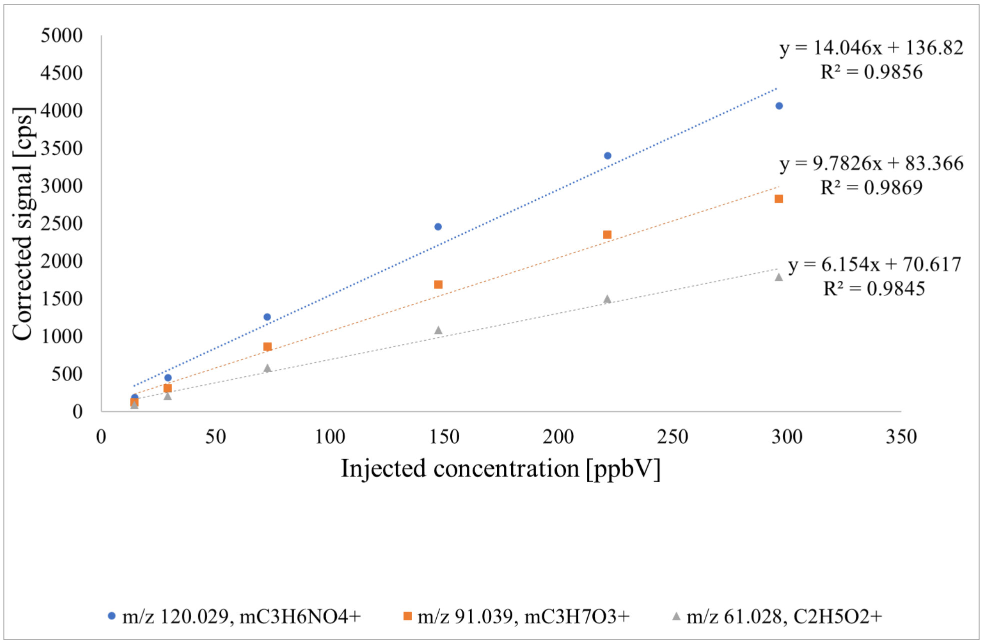

Figure S7. Calibration plot of response (cps) versus concentration (ppbV) for glycerin in NO^+ mode (E/N 16 Td). *NB: a second injection was performed mid-range for quality control purposes to verify the consistency of response.*

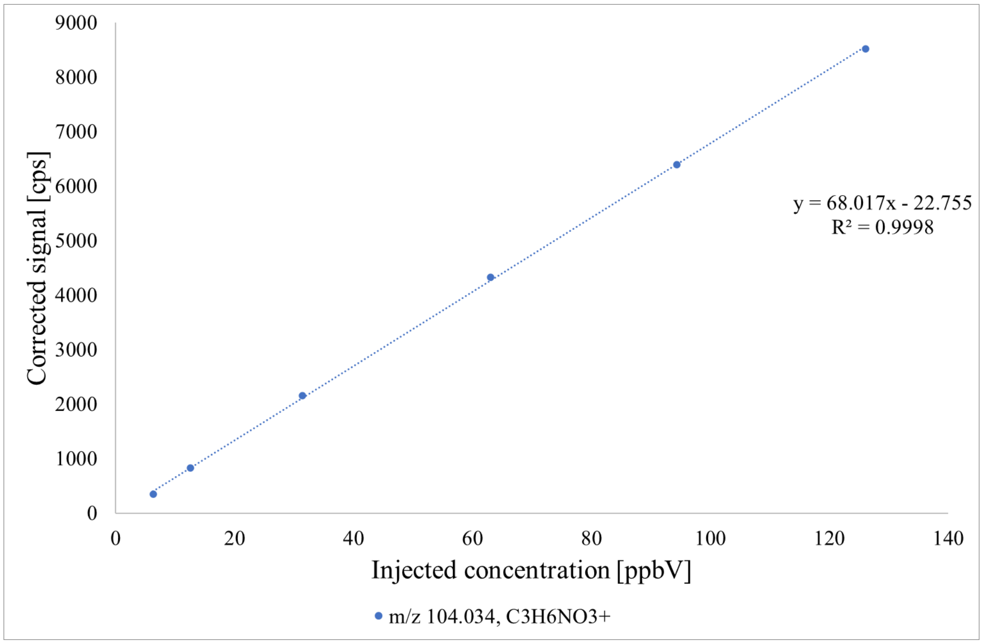

Figure S8. Calibration plot of response (cps) versus concentration (ppbV) for acetol in NO^+ mode (E/N 16 Td). *NB: a second injection was performed mid-range for quality control purposes to verify the consistency of response.*

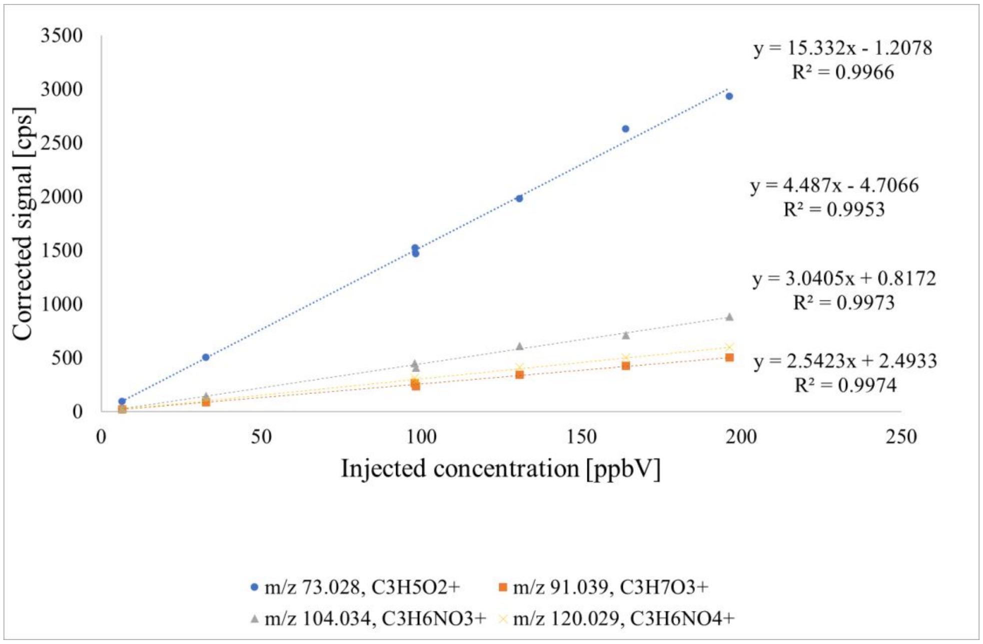

Figure S9. Calibration plot of response (cps) versus concentration (ppbV) for glycidol in NO^+ mode (E/N 16 Td). *NB: a second injection was performed mid-range for quality control purposes to verify the consistency of response.*

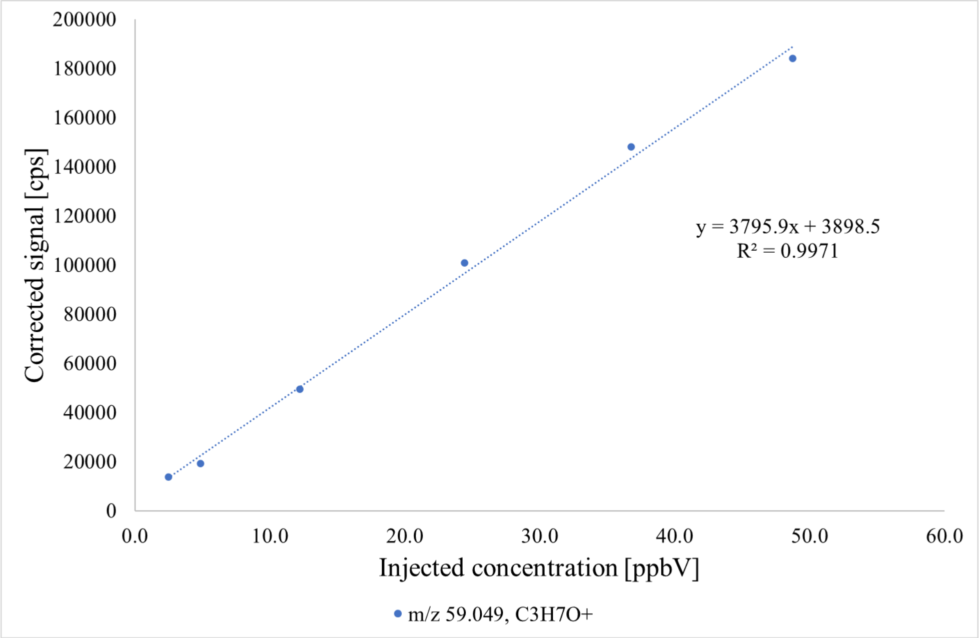

Figure S10. Calibration plot of response (cps) versus concentration (ppbV) for acetone in H_3O^+ mode (E/N 69 Td). *NB: a second injection was performed mid-range for quality control purposes to verify the consistency of response.*

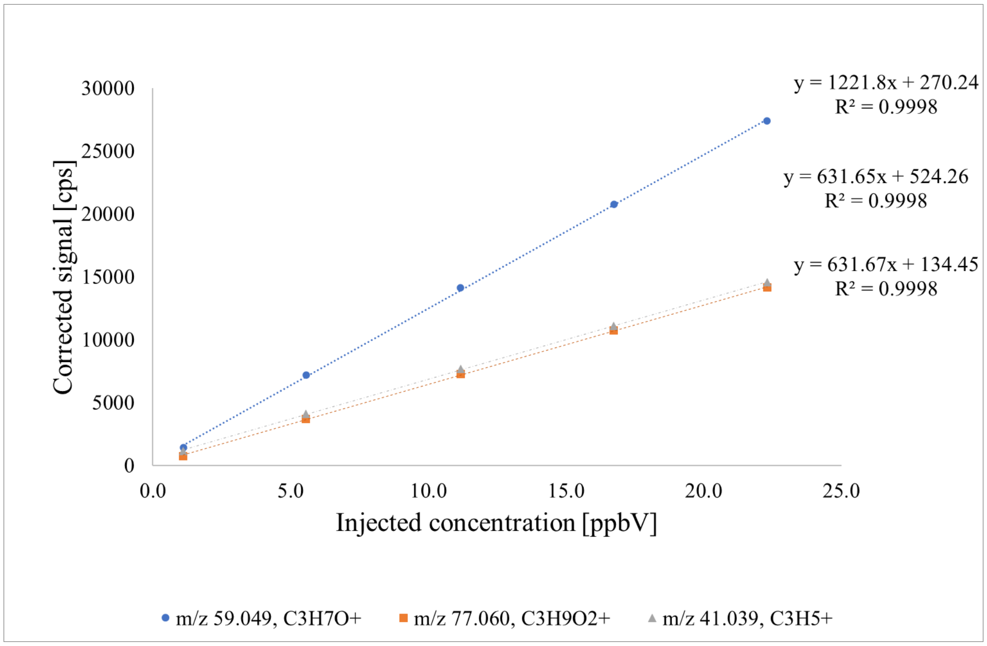

Figure S11. Calibration plot of response (cps) versus concentration (ppbV) for propylene glycol in H_3O^+ mode (E/N 69 Td). *NB: a second injection was performed mid-range for quality control purposes to verify the consistency of response.*

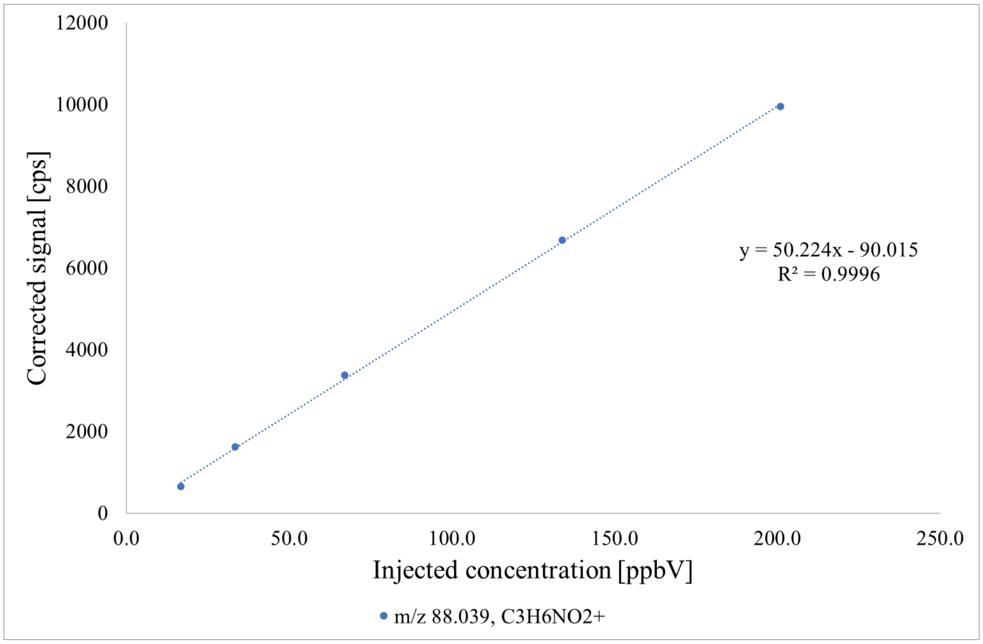

Figure S12. Calibration plot of response (cps) versus concentration (ppbV) for acetone in NO^+ mode (E/N 16 Td). *NB: a second injection was performed mid-range for quality control purposes to verify the consistency of response.*

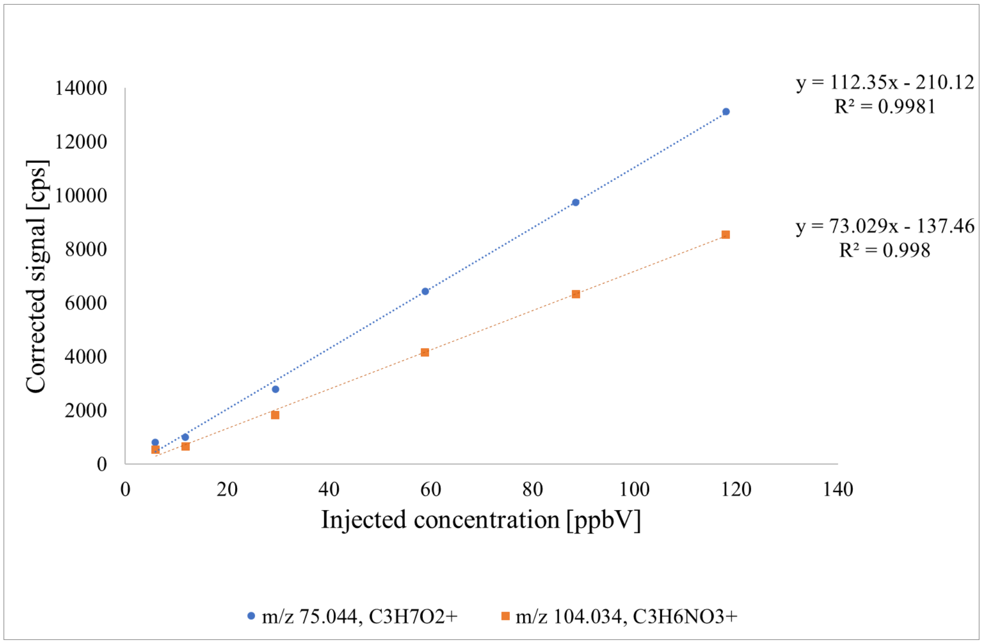

Figure S13. Calibration plot of response (cps) versus concentration (ppbV) for propylene glycol in NO^+ (E/N 16 Td). *NB: a second injection was performed mid-range for quality control purposes to verify the consistency of response.*

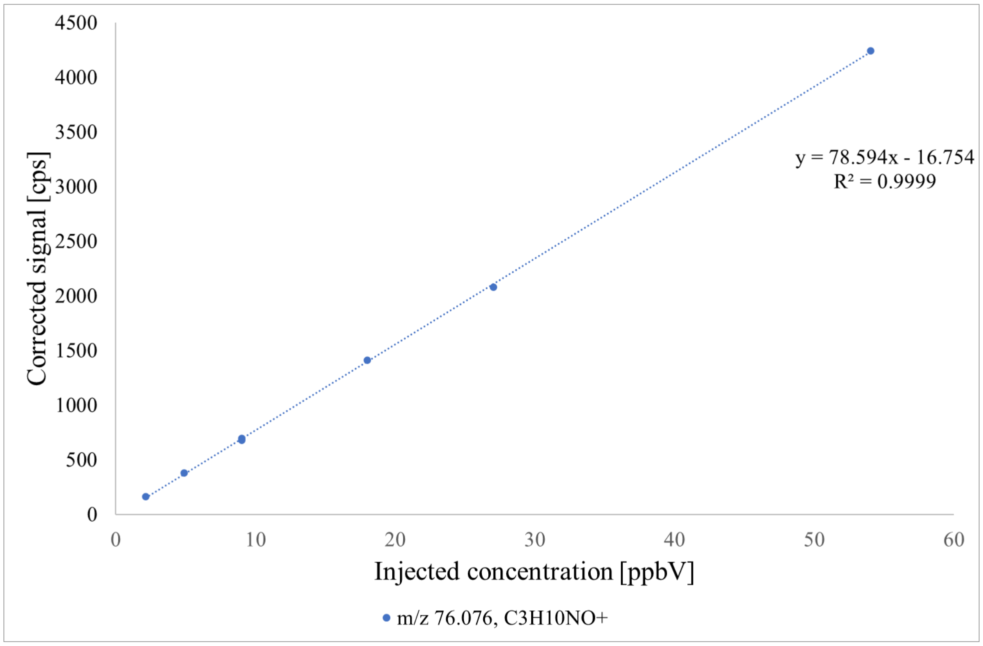

Figure S14. Calibration plot of response (cps) versus concentration (ppbV) for acetone in NH_4^+ mode (E/N 36 Td). *NB: a second injection was performed mid-range for quality control purposes to verify the consistency of response.*

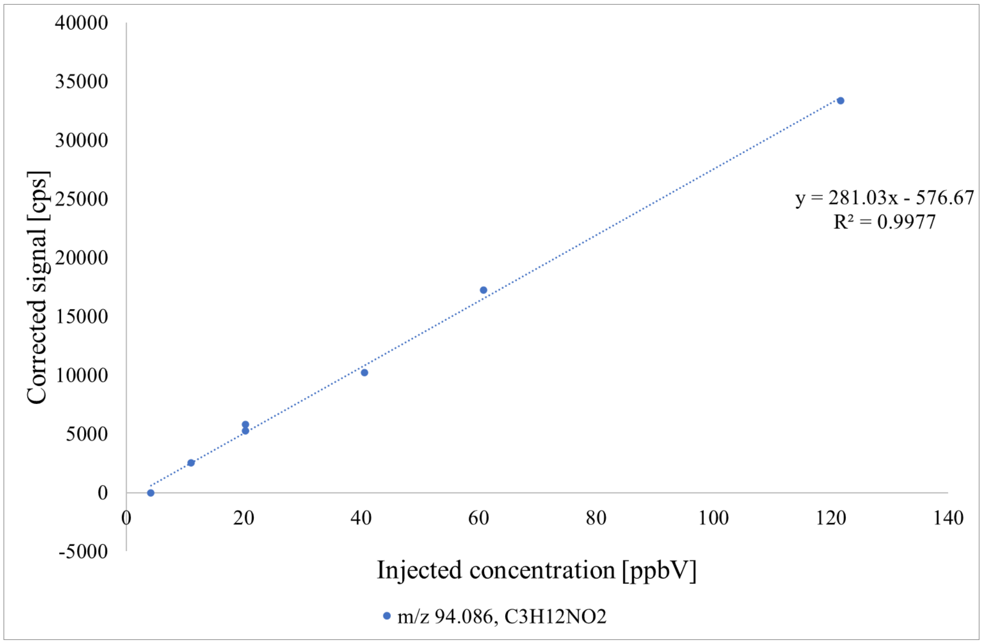

Figure S15. Calibration plot of response (cps) versus concentration (ppbV) for propylene glycol in NH_4^+ mode (E/N 36 Td). *NB: a second injection was performed mid-range for quality control purposes to verify the consistency of response.*
